# Neuronal excitatory-to-inhibitory balance is altered in cerebral organoid models of genetic neurological diseases

**DOI:** 10.1186/s13041-021-00864-w

**Published:** 2021-10-11

**Authors:** Simote T. Foliaki, Benjamin Schwarz, Bradley R. Groveman, Ryan O. Walters, Natalia C. Ferreira, Christina D. Orrù, Anna Smith, Aleksandar Wood, Olivia M. Schmit, Phoebe Freitag, Jue Yuan, Wenquan Zou, Catharine M. Bosio, James A. Carroll, Cathryn L. Haigh

**Affiliations:** 1 Laboratory of Persistent Viral Diseases, Rocky Mountain Laboratories, National Institute of Allergy and Infectious Diseases, National Institutes of Health, Hamilton, MT 59840 USA; 2 Laboratory of Bacteriology, Rocky Mountain Laboratories, National Institute of Allergy and Infectious Diseases, National Institutes of Health, Hamilton, MT 59840 USA; 3 Departments of Pathology and Neurology, Case Western Reserve University School of Medicine, Cleveland, OH 44106 USA

**Keywords:** Neurodegenerative diseases, Neuronal network communication, Neural oscillation

## Abstract

The neuro-physiological properties of individuals with genetic pre-disposition to neurological disorders are largely unknown. Here we aimed to explore these properties using cerebral organoids (COs) derived from fibroblasts of individuals with confirmed genetic mutations including *PRNP*^*E200K*^, trisomy 21 (*T21)*, and *LRRK2*^G2019S^, which are associated with Creutzfeldt Jakob disease, Down Syndrome, and Parkinson’s disease. We utilized no known disease/healthy COs (*HC*) as normal function controls. At 3–4 and 6–10 months post-differentiation, COs with mutations showed no evidence of disease-related pathology. Electrophysiology assessment showed that all COs exhibited mature neuronal firing at 6–10 months old. At this age, we observed significant changes in the electrophysiology of the COs with disease-associated mutations (dCOs) as compared with the *HC*, including reduced neuronal network communication, slowing neuronal oscillations, and increased coupling of delta and theta phases to the amplitudes of gamma oscillations. Such changes were linked with the detection of hypersynchronous events like spike-and-wave discharges. These dysfunctions were associated with altered production and release of neurotransmitters, compromised activity of excitatory ionotropic receptors including receptors of kainate, AMPA, and NMDA, and changed levels and function of excitatory glutamatergic synapses and inhibitory GABAergic synapses. Neuronal properties that modulate GABAergic inhibition including the activity of Na–K-Cl cotransport 1 (NKCC1) in Cl^−^ homeostasis and the levels of synaptic and extra-synaptic localization of GABA receptors (GABARs) were altered in the *T21* COs only. The neurosteroid allopregnanolone, a positive modulator of GABARs, was downregulated in all the dCOs. Treatment with this neurosteroid significantly improved the neuronal communication in the dCOs, possibly through improving the GABAergic inhibition. Overall, without the manifestation of any disease-related pathology, the genetic mutations *PRNP*^*E200K*^, *T21*, and *LRRK2*^G2019S^ significantly altered the neuronal network communication in dCOs by disrupting the excitatory-to-inhibitory balance.

**Supplementary Information:**

The online version contains supplementary material available at 10.1186/s13041-021-00864-w.

## Background

Neurodegenerative diseases are a leading cause of morbidity and mortality, and their incidence is expected to increase as populations live to older ages. These diseases include Alzheimer’s Disease (AD), Parkinson’s Disease (PD), and prion diseases such as Creutzfeldt Jakob Disease (CJD). A common feature of these diseases is accumulation of disease-associated protein deposits within the brain leading to eventual dysfunction and death. However, the precise mechanisms by which these proteins cause dysfunction remain unknown. Research into these diseases has been limited by the availability of live human neuronal cultures for studying how these diseases cause dysfunction and death in the human brain. The recent development of cerebral organoid (CO) cultures [1–3], differentiated from human induced pluripotent stem cells (iPSCs), allows human neuronal tissue to be available on demand for such studies.

COs have been used to study a variety of diseases and infections of the brain [4, 5]. COs generated from familial Alzheimer’s Disease (AD) donors show the accumulation of the AD-associated amyloid beta protein [6]. This is also observed for COs from Down Syndome (DS)/Trisomy 21 (*T21*) donors where AD pathology occurs in the brain approximately 50% of patients due to the overexpression of the amyloid precursor protein, which is located on chromosome 21 [6]. Organoid models of sporadic PD caused by *LRRK2*^G2019S^ mutation exhibit increased levels of alpha-synuclein oligomers with increased vulnerability to cell death caused by 1-methyl-4-phenyl-1,2,3,6-tetrahydropyridine [7]. Additionally, our work has previously shown that COs can model infectious prion disease induced by exposure to brain homogenate from patients who died of sporadic CJD [5]. The development of these new human models allows for investigation of neuronal functions and degeneration in detail that has not previously been possible.

Neuronal function is changed in many neurodegenerative diseases. Specific receptors and signalling pathways linked with DS, PD, CJD include glutamate receptors known to bind N-Methyl-D-aspartic acid (NMDARs), kainite receptors (KARs), and α-amino-3-hydroxy-5-methyl-4-isoxazolepropionic acid receptors (AMPARs). NMDARs are essential for neuronal excitation associated with memory formation; however hyperactive NMDARs often disrupt synaptic activity and lead to excitotoxicity and neurodegeneration [8, 9]. AMPARs and KARs are structurally similar and known for their rapid channel gating in response to agonists [10], which allows them to play essential roles in the maintenance of neuronal excitability. Another type of receptor known to change in neurological disorders is gamma-Aminobutyric Acid receptors (GABARs). GABARs are vital components of the neuronal inhibitory system that maintains optimal levels of neuronal excitation. Reduced activity of GABARs leads to the neuronal hyperexcitability implicated in epileptic seizure [11], while hyperactive GABARs prevent neuronal excitability [12]. The function of GABARs is dependent on the intracellular Cl- gradient, which is dependent on the roles of Na–K–Cl cotransporter 1 and 2 (NKCC1 and NKCC2) in allowing Cl^−^ influx and efflux. Overexpression of NKCC1 correlates with GABAR becoming excitatory in experimental models of some neurological disorders including Down syndrome [13, 14]. Evidence has shown that NKCC1 activity is excessive in immature and abnormal neurons, thus triggering the GABARs to depolarize neurons and increase neuronal excitability associated with seizure [13, 15]. The mixed population of neurons found within COs now allows to study these proteins and pathways in a live human 3D tissue.

The CO model provides a new opportunity to investigate the electrophysiology of human neuronal tissue in vitro. COs develop similarly to human brain tissue, with demonstrable electrophysiological functions that develop mature and more complex networks after approximately 6 months [2, 3]. The neuronal receptors and ion channels are functional, forming synapses and intricate signalling pathways in COs [2, 3, 16]. Astrocytes and oligodendrocytes also develop within COs between approximately months 3 and 5 [17], likely contributing to the neuronal activity and network complexity [2, 3, 16]. Neuro-electrophysiology analyses of COs from familial AD have established that abnormal function in association with AD pathology can be characterized [18]. This use of neuro-electrophysiology to examine the CO neuronal networks permits both developments of a better understanding of neurodegenerative disease processes and of how mutations within genes associated with neurodegeneration influence neuronal function.

While we have previously shown that COs can model infectious prion disease, we and others also found that COs generated from donors with the E200K mutation within the prion protein gene (*PRNP*) that causes genetic CJD do not show any parameters of disease [6, 19]. To investigate whether the *PRNP*^*E200K*^ mutation can influence electrophysiological functions of the CO cultures and contrast this with the trisomy 21-causing AD and *LRRK2*^G2019S^ -causing PD mutations, organoids at 3–4 and 6–10 months post differentiation were monitored for electrophysiological aberrations and for changes in their synaptic receptors.

## Methods

### ATCC human induced Pluripotent Stem Cells (hiPSCs)

Four lines of hiPSCs were purchased from the American Type Culture Collection (ATCC). Two lines, ATCC­HYS0103 (ATCC® ACS­1020™) and KYOU­DXR0109B [201B7] (ATCC® ACS­1023™), were classified as ‘normal’ (no known neurodegenerative disease), ATCC­DYP0730 (ATCC® ACS­1003™) were generated from a donor with Down Syndrome (*T21*), and ATCC-DYS0530 (ATCC® ACS-1013™) were generated from a donor with *LRRK2*^G2019S^ mutation causing Parkinson’s disease.

### Generation of E200K(1), E200K(2) and RAH019A hiPSCs

Two asymptomatic *PRNP*^*E200K*^ donor iPSC lines and a further no known disease control iPSC line were made from donor fibroblasts as described previously [19]. Briefly, fibroblasts were collected by skin punch and grown in DMEM supplemented with 10% (v/v) fetal bovine serum, 1 × glutamax and penicillin/streptomycin. Reprogramming was achieved using ReproRNA™-OKSGM and ReproTeSR™ medium (Stemcell Technologies) as per the manufacturer’s instructions.

### hiPSC culture

Human iPSC lines were routinely cultured in low growth factor Matrigel (Roche) in mTeSR1 medium (Stem Cell Technologies) with 5% CO2 in a humidified incubator. Media was changed daily and colonies were passaged at approximately 70–80% confluency before contact between colonies could occur, as previously described in [5, 19].

### Organoid generation and culture

Organoids were generated and maintained as described previously [5, 19]. Organoids were routinely cultured in complete maintenance medium (1 × glutamax, 1 × penicillin–streptomycin solution, 0.5 × non-essential amino acids, 0.5% [v/v] N2, 1% (v/v) B12 with retinoic acid, 0.025% (v/v) insulin, and 0.00035% (v/v) 2-Merceptoethanol in 1:1 Neurobasal:DME-F12 medium), under standard incubator conditions (5% CO2, 37 °C, humidified), on an orbital shaker at 85 rpm using vented conical flasks (Corning).

### Neuro-electrophysiology

COs were adapted to the Brainphys media (recording media) by incubating them in 50:50 Brainphys media (StemCell Technologies): complete maintenance media for two days and in 70:30 Brainphys media: complete maintenance media for another two days before recording their electrophysiology in 100% Brainphys media at 32 °C. Harp slice grids (Multichannel Systems) were used to submerge the organoids and ensure that they firmly contacted the electrodes. For assessing the baseline neuronal firing/oscillation at 6–10 months old, we used COs from multiple batches differentiated at different times. The synchronous neuronal population activities were detected using Multielectrode arrays (MEA) embedded with 60 titanium platinum microelectrodes (8 × 8 layout; Electrode spacing 200 µm; Electrode diameter 30 µm; one internal reference electrode) in a MEA2100-System with an integrated amplifier and recorded by McRack software (Multichannel Systems). The raw data were sampled at 25000 Hz and filtered by a Savitxky-Golay FIR filter (second order and 8 points). The basal neuronal population functions were recorded for at least 5 min to ensure stabilization before sequentially applying increasing doses of pharmacological treatments (for 5 min per dose). Between doses was a minute washout. These treatments included glycine with NMDA (in the absence of glutamate; Abcam), Kainate (Abcam), TTX (1 µM), AMPA (Abcam), and GABA(Abcam). The raw, filtered, and spike data were exported for further analyses. The neuronal network communication was abolished by the sodium channel blocker tetrodotoxin (Additional file 1a).

### MEA data analyses

For the detection of the neuronal population firings, the signals were filtered by a High-pass filter (300–2500 Hz) and the peaks or spikes were detected as those with amplitudes greater than the 4 standard deviations of the mean, calculated for each electrode in the first 30 s of the reading. The filtered and spike data were further analysed by a MATLAB toolbox, MEAnalyzer, to determine the spike rate, burst rate, network connectivity, and the periodicity of the spike rate and network events [20]. Electrodes or channels with less than or equal to 12 spikes per a minute were inactive channels. A burst was defined as at least 4 spikes in 100 ms. The periodicity of the network events (based on the spiking percentage) was calculated by Autocorrelation and Welch’s Periodogram. The detection threshold was defined as the peak of normalized autocorrelation/power above one standard deviation of the mean [20]. The connectivity based on the spike correlation was defined as two channels with a spiking correlation coefficient above 0.5 [20]. Each parameter was recorded from all the active electrodes (except the reference electrode) and averaged to represent an organoid. As recommended in order to maximize the accuracy of the algorithm used to calculate the network connectivity [20], we only used a minute timeframe for the data analysis, which was randomly selected from the ~ 5 min recording time.

The raw data were processed as described previously [2], by filtering with a low-pass FIR filter (< 1000 Hz) and down sampling from 25,000 to 1000 Hz (*resample.mat*) for the neural oscillation analyses. For the narrow-band relative oscillatory power, the raw data were transformed into time–frequency domain by a continuous wavelet synchrosqueezing transformation (*wsst.mat)*, decomposed into narrow-band frequency ranges (delta: < 4 Hz, theta: 5–8 Hz, alpha: 9–13 Hz, beta: 14–32 Hz, low gamma 33–80 Hz, upper gamma: 100–200 Hz) by inverse wsst (*iwsst.mat*). The oscillatory power of each narrow-band frequency range was estimated by the Welch’s method (*pwelc*.*mat)* with a window length of 2000 ms and overlap of 1000 ms. For the oscillatory power connectivity, the relative oscillatory power of each narrow band in every channel was calculated in every 5 s for five minutes and the correlation between channels based on their oscillatory power was calculated by Pearson’s correlation. Those electrodes with correlation coefficients above one standard deviation from the median (with > 90% confident interval) were significantly connected. The Pearson’s correlation coefficients measured the weights of connectivity. We computed the modulation index of the phase-amplitude coupling between neural oscillations as described previously [21, 22]. MATLAB codes can be found in Additional files 2, 3, 4, 5, 6, 7.

### Immunohistochemistry

As published previously [4], cerebral organoids were fixed in 10% (v/v) formalin for 24 h at room temperature, washed with 1xPBS, incubated in 20–30% sucrose for 24 h at room temperature, embedded in OCT, and frozen at 20 °C until slicing. The surface AMPARs and GABARs were labelled with LiveReceptor AMPAR (Funakoshi, Cat No.: FDV-0018A) and GABAR (Funakoshi, FDV-0018B) [23] by incubating in 1 µM of LiveReceptor for 3 h at room temperature before fixing in 10% (v/v) formalin. The frozen organoids were sliced into ~ 10 µm thick slices using a cryostat (LEICA CM 3050 S). The slices were blocked for an hour at room temperature in a blocking solution containing 5% (w/v) BSA, 0.3 M glycine (formalin quenching solution), and 0.1% Triton X-100 (permeabilizing solution). The slices were then labelled with primary antibodies (Additional file 8) against the proteins of interest. DAPI stain (concentration of 290 nM) was used to label cells. The immuno-labelled proteins were detected by Alexa Fluor 488-, 555-, and 647-labelled secondary antibodies (Invitrogen). The LiveReceptor labels were detected by anti-fluorescein dye (Alex Fluor 488) antibody (Cat No. A-11090, Invitrogen). Images were taken using an EVOS-FL-auto light microscope (Invitrogen) at 4× and 20× magnification and Confocal microscope (Zeiss laser scanning LSM 880 microscope driven by ZEN v.2.3 software). We used Huygens Essential 20.04 software to calculate protein colocalization (Pearson’s coefficient). We used ImageJ (1.52i) to quantify the average fluorescent intensity of neuronal markers on the whole organoid section imaged at 4× magnification. The quantification was limited to regions that were DAPI positive to avoid processing areas devoid of cells. We removed the background fluorescence by subtracting the fluorescence, measured in tissue sections stained with secondary antibodies only, from the marker’s fluorescence.

### Calcium assay

The intracellular calcium was measured by Fluo-4 Direct Calcium Assay Kit (F10471). Organoids were plated into 96-well plate (one organoid per well) wherein each well contained 50 µL of A+ media. 50 µL of 2 × Fluo-4 Direct calcium reagent was added into each well. The fluorescence (excitation at 494 nm and emission at 516 nm) was measured every minute in a circular pattern for an hour at 37 °C. The total protein levels of each organoid were measured by BCA assay after lysing each organoid in 1 × RIPA buffer and diluting by 10 times in 1xPBS. The estimate of the intracellular calcium levels was obtained by normalizing the mean fluorescence for individual organoids by their total protein levels.

### RT-PCR

For quantitative analysis of changes in transcription from 6-month-old human cerebral organoids, 400 ng of high-quality RNA from each sample was reverse transcribed to synthesize cDNA using the RT2 First Stand Kit per manufacturer’s instructions (Qiagen). Each cDNA reaction was mixed with 2× RT2 SYBR Green Mastermix purchased from Qiagen with RNase-free water to a final volume of 1.3 ml. Ten microliters of the mixture was then added to each well of a 384-well format plate of the Human Neurotransmitter Receptor Array PAHS-060ZE (Qiagen).

The analysis was carried out on an Applied Biosystems ViiA 7 Real-Time PCR System with a 384-well block using the following conditions: 1 cycle at 10 min, 95 °C; 40 cycles at 15 s, 95 °C then 1 min, 60 °C with fluorescence data collection. Melting curves were generated at the end of the completed run to determine the quality of the reaction products. Raw threshold cycle (CT) data was collected with a CT of 35 as the cutoff. CT data was analyzed using the web-based RT^2^ Profiler PCR Array Data Analysis from Qiagen. All CT values were normalized to the geometric mean of the CT values for the housekeeping genes ACTB, GAPDH, HPRT1, and RPLP0. Changes in transcription were calculated by the software using the ΔCT based method [24]. Statistical analysis was performed using the unpaired Student’s t-test to compare the replicate ΔCT values for each gene in the control group versus experimental groups. A mean of ≥ or ≤ a 2.0-fold change and p-value of ≤ 0.05 were considered significant. For qRT-PCR data the p-values were not adjusted for multiple comparisons since we were interested in only controlling for the individual error rate, where an adjustment for multiple tests is deemed unnecessary. A Pearson correlation heatmap and average linkage hierarchical cluster analysis was generated using the -ΔCT (housekeeping gene CT- experimental gene CT) values for each using the web-enabled application Heatmapper [25].

### Neurotransmitter analysis

#### Materials

Tributylamine was purchased from Millipore Sigma. LCMS grade water, methanol, isopropanol, chloroform, acetonitrile, formic acid and acetic acid were purchased through Fisher Scientific. All standards were purchased from Sigma.

### Sample processing

For all LCMS methods, LCMS grade solvents were used. Sample order was randomized throughout each extraction. For media samples, macromolecular components were precipitated by addition of two volumes of ice-cold methanol to one volume of media. Samples were incubated at −20 °C for 1 h and subsequently centrifuged at 16,000×*g* for 20 min at 4 °C to pellet precipitate. The supernatant was taken directly for LCMS analysis. For organoid samples, a single organoid was submerged in 500 µL of ice-cold methanol and homogenized through a screen. The homogenate was collected and 500 µL each of ice-cold water and chloroform was added. The sample was agitated for 20 min at 4 °C by shaking and then spun at 16,000×*g* for 20 min at 4 °C to induce layering. The top (aqueous) layer was collected. The aqueous layer was diluted 1:5 in 50% methanol in water and prepared for LCMS injection.

### LC–MS/MS analysis

All molecular analysis was performed using a series of targeted multiple-reaction monitoring (MRM) methods. All samples were separated using a Sciex ExionLC™ AC system and analyzed using a Sciex 5500 QTRAP® mass spectrometer.

For gamma aminobutyric acid (GABA), norepinephrine, dopamine, serotonin, N-acetylaspartylglutamic acid (NAAG), and acetylcholine spectra were collected, and candidate multiple reaction monitoring (MRM) pairs were established from the most abundant fragment peaks (Additional file 9). The uniqueness of each potential MRM was evaluated by spiking standard into sample matrix consisting of a quality control mixture of either media or organoid sample. All other MRMs were from previously published methodology [26].

Relative quantification was performed using quality control sample injections after every 10 injections and assessed for signal stability. MRM fidelity was confirmed by comparison of retention time to standards and collection of triggered spectra. For negative mode targets, samples were separated across a Waters Atlantis T3 column (100 Å, 3 µm, 3 mm × 100 mm) and eluted using a binary gradient from 5 mM tributylamine, 5 mM acetic acid in 2% isopropanol, 5% methanol, 93% water (v/v) to 100% isopropanol over 15 min. For positive mode targets, samples were separated across a Phenomenex Kinetex F5 column (100 Å, 2.6 µm, 100 × 2.1 mm) and eluted with a gradient from 0.1% formic acid in water to 0.1% formic acid in acetonitrile over 5 min. All samples were analyzed using both methods as part of broader panel of metabolites.

When possible, targets were detected by two or more MRM pairs per target. Compatible targets including arginine, aspartate, glutamate, adenosine, GABA, Serine, NAD+, NADH, tryptophan and tyrosine were detected in both methods to confirm agreement (Additional file 10). MRM data was filtered using a missing value cutoff for each sample set of 50% and a coefficient of variance for the QC injections of 30%. The z-score was calculated for each neurotransmitter across organoid lines using GraphPad Prism 8.

### Tau RT-QuIC analysis

The purification of K12CFh substrate was performed as previously described [27]. The K12 RT-QuIC assay was performed as follows: 6.5 μM–0.1 mg/mL of K12CFh substrate was filtered through 100 kDa filters and added to a reaction mix containing 40 mM HEPES (pH 7.4), 400 mM NaF (buffered in 10 mM HEPES, pH 7.4), 40 μM heparin, and 10 μM thioflavin T (ThT). Organoid homogenates (10% w/v in PBS) were serially diluted in tenfold steps in a dilution buffer containing 0.53% tau-free mouse brain homogenate (KO; tauGFP from Jackson Laboratories) and 1 × N2 Supplement (Gibco) in 10 mM HEPES. Forty-eight microliters of reaction mix were added in each well of a 384-well plate followed by the addition of 2 µL of specified organoid dilution. Each organoid dilution was assessed in quadruplicate in a 384-well optically clear bottom plate (NUNC). Plates were sealed and inserted into an Omega FLUOStar plate reader and subjected to the following conditions: 1 min shaking, and 1 min rest, 500 rpm, orbital, at 42 °C, with ThT fluorescence reads (450 excitation, 480 emission) taken every 15 min.

### α-synuclein RT-QuIC analysis

The K23Q α-synuclein substrate was purified, and the assay was run as previously described [28]. Brain and organoids were homogenized to 10% in PBS and cleared with a brief 2000×*g* 2 min centrifugation. Samples were considered positive for seeding activity if the fluorescent signal for ≥ 50% of the replicate wells exceeded 10% of the maximum value on the plate prior to 40 h.

### Detergent insolubility assay and Western blot

We adapted the detergent insolubility assay from a previously published protocol [7]. Briefly, lysates (20 μl of 10% w/v in RIPA lysis buffer) were treated with 300 μl of 10% (w/v) sarkosyl for 1 h at RT with constant agitation at 1400 rpm, diluted with 2680 μl of H-Buffer, and centrifuged at 100,000×*g* for 1 h at 4 °C. The pellets were resuspended in 1 × sample buffer for western blot analysis. The proteins in the supernatants were precipitated by methanol precipitation as published previously [19], and resuspended in 1 × sample buffer for western blot analysis. Protein preparations (lysates or brain homogenates in 1 × RIPA buffer) were denatured by boiling in 1 × sample buffer (containing 5% v/v Beta-mercaptoethanol) for 5 min. Denatured proteins were resolved in SDS-PAGE and transferred into PVDF membrane for immunoblotting. The membrane was blocked with 5% (w/v) blocking solution (ThermoFisher) for 1 h and labelled for Abeta/APP and Tau by 6E10 antibody (Additional file 8) and AT8 antibody (Additional file 8). The immuno-labelled proteins were detected with anti-mouse HRP (Abcam) and visualized by ECL Select (Amersham) and imaged on the iBright imaging system (Invitrogen). The total protein was stained with a No-stain protein labelling reagent (Invitrogen).

### Data analysis and statistics

Formal statistical analyses were performed in Prism 8.2.0 and MATLAB R2020a. Data were tested for conformity to a Gaussian distribution of residuals and for the presence of statistical outliers (ROUT test, Q = 1%). If not stated otherwise, one-way ANOVA or one-way ANOVA by ranks (Kruskal–Wallis), with the appropriate secondary test was used to compare the neuro-electrophysiological features, synaptic properties, and neurotransmitters of dCOs and *HC*. Repeated measures (RM) One-way ANOVA was used to compare age-dependent change in the neurotransmitters of dCOs and *HC*. RM Two-way ANOVA was also used to compare the change in burst rate and intracellular calcium in response to increasing doses of the agonists of ionotropic receptors.

## Results

### Assessment of neuropathological features in the COs with disease-associated mutations (dCOs)

We first examined the *PRNP*^E200K^, *LRRK2*^G2019S^ and *T21* organoids for any pathology associated with their respective human diseases. We previously reported that both lines of *PRNP*^E200K^ organoid do not display any pathology associated with CJD at 6 and 12 months old [19]. Pathological features of DS that are known to resemble those of AD, including the accumulation of amyloid beta (abeta42 fragment) and insoluble Tau aggregation and RT-QuIC seeding activity [29], were not evident in the *T21* COs (Additional file 11a, e, f, g) [29]. We also found no evidence of the typical pathological correlates of PD, alpha-synuclein accumulation and rapid αSyn RT‐QuIC (αSyn RT‐QuICR) seeding activity, in the *LRRK2*^G2019S^ COs (Additional file 11b, h). Given the direct involvement of prion protein (PrP) in the pathogenesis of some neurological disorders [30], we measured the expression of PrP and found no change in the dCOs compared with the *HC* (Additional file 11c). No COs displayed protein aggregation that could be detected by the Thioflavin T (ThT) probe (Additional file 11d). The dCOs showed normal numbers of active astrocytes, suggesting no disease-associated astrogliosis in these organoids (Additional file 11i). Overall, none of the dCOs exhibited measurable pathology associated with their respective clinical disease in humans although it is possible that pathology was present but below the limits of detection.

### dCOs exhibited weaker neuronal network communication

**Fig. 1:**
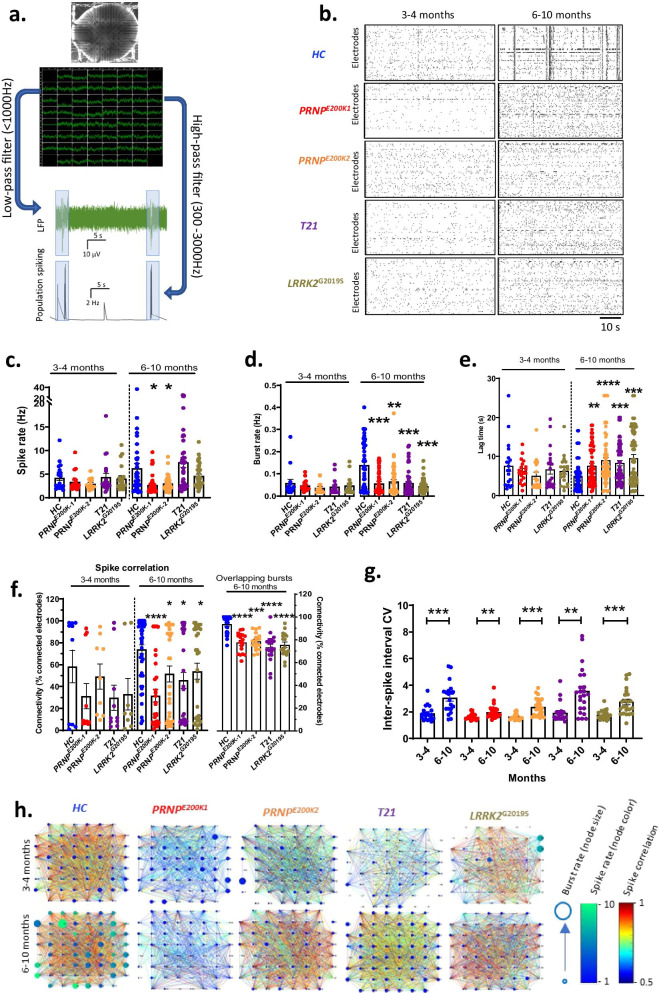
Neuronal firing and network communication at 3–4 and 6–10 months old. **a** Top panel: a picture of cerebral organoids on Multi-electrode arrays (MEA) with the black dots indicating the electrodes; Middle panel: a representative diagram of local field potential detected by each electrode shown in the top panel); Bottom panel: a diagram displaying how the raw data were processed. **b** Representative raster blots displaying neuronal firings or spikes (dots), bursts (clusters of dots), and network communication (depicted by electrodes with overlapping bursts) in the healthy controls (*HC*) and the organoids with genetic defects (*PRNP*^*E200K1*^, *PRNP*^*E200K2*^, *T21*, and *LRRK2*^*G2019S*^). **c** Spike rate (n = 18 to 20 at 3–4 months; n = 36 to 44 at 6–10 months). **d** Burst rate (n = 14 to 19 at 3 months; n = 50 at 6–10 months). **e** Periodicity of network firing (n = 18 to 20 at 3–4 months; n = 62 to 67 at 6–10 months). **f** Percentage of connected electrodes based on spike correlations (left panel; n = 8 to 11 at 3–4 months; n = 30 to 46 at 6–10 months) and overlapping bursts (right panel; n = 17 to 29 at 6–10 months). **g** Inter-spike interval coefficient of variation (CV; n = 18 to 20 at 3–4 months; n = 20 to 23 at 6–10 months). **h** Representative graphs of the connectivity between electrodes (nodes) based on the spike correlation with the node colour and size representing the burst rate and spike rate. **c**–**f** The parameters of neuronal spiking in the organoids with mutations were compared to the age-matched *HC* by One-way ANOVA on ranks with Dunnett’s correction for multiple comparisons. **g** Paired Student’s t-test on ranks was used to analyse the age-dependent change in the inter-spike interval CV. Each point on the graphs represents an individual organoid. If not otherwise indicated CO colour code are as follows; *HC* (blue), *PRNP*^*E200K−1*^ (red), *PRNP*^*E200K−2*^ (yellow), *T21* (purple) and *LRRK2*^*G2019S*^ (grey). Bars and error denote mean and SEM. * p < 0.05, **p < 0.01, ***p < 0.001, ****p < 0.0001

We aimed to determine if the genetic mutations altered neuronal electrical signaling and network communication in the absence of disease pathology. Previous studies showed that COs reach maturity around 6 months old in terms of their neuronal oscillation and complementation of cell types, including astrocytes and oligodendrocytes [2]. We assessed the network communications between populations of neurons in the dCOs, by measuring the local field potential (LFP) using multi-electrode arrays (Fig. 1a) at 3–4, 6–7 and 8–10 months old to determine any functional dysregulation compared with healthy control (*HC*, no known disease) COs. We extracted the neuronal population firing by a high-pass filter (Fig. 1a) and determined the neuronal population bursting, periodicity of network events, and the connectivity between networks of neurons. Organoids in the 6–7 and 8–10 groups demonstrated equivalent activities, with no significant differences measured between these ages (Additional file 1d, e); therefore, these readings have been grouped together and presented as a 6–10-month-old group. Likewise, no significant differences were measured between control donors (Additional file 1f) and so the three *HC* donor lines are grouped together to produce a more representative reference range for normal organoid function and for ease of interpretation. At 3–4 months old, the dCOs produced neuronal population firings (spikes) at rates that were not different from the *HC* (Fig. 1b, c). At this age, neither the burst rate and periodicity of network events, which measured the neuronal network communication, (Fig. 1d, e) nor the connectivity of the channels (the percentage of connected channels based on spike correlation) were different in the dCOs from the *HC* (Fig. 1f, h).

We next evaluated the changes as the organoids matured after 6 months. As expected, the coefficient of variation (CV) of neuronal population firing rate was significantly increased at 6–10 months relative to 3–4 months in all COs (Fig. 1g), replicating the previously reported increase in neuronal function in COs with age [2]. At this later timepoint, the *PRNP*^*E200K1*^ and *PRNP*^*E200K2*^ organoids, but not the *T21* and *LRRK2*^*G2019S*^, produced a significantly lower spike rate than the *HC* (Fig. 1b*, *c). The neuronal population network communication was significantly lower in all the dCOs as demonstrated by the slower burst rate (Fig. 1d), longer periodicity of network events (Fig. 1e), and weaker connectivity of the channels as estimated by the percentage of connected channels based on spike correlation and overlapping bursts (Fig. 1f, h). The weight (strength of the connection between two nodes) of the connectivity based on spike correlation were not different in the dCOs (Additional file 1b). Further, we found no significant increase in the intracellular calcium levels of these organoids to suggest any excitotoxicity associated with the altered neuronal population communication (Additional file 1c). Overall, relative to the *HC* COs, the neuronal population communication was largely normal in all the dCOs at 3 months, but it became significantly weaker after 6 months old.

### Altered neural oscillations occur in the dCOs

**Fig. 2:**
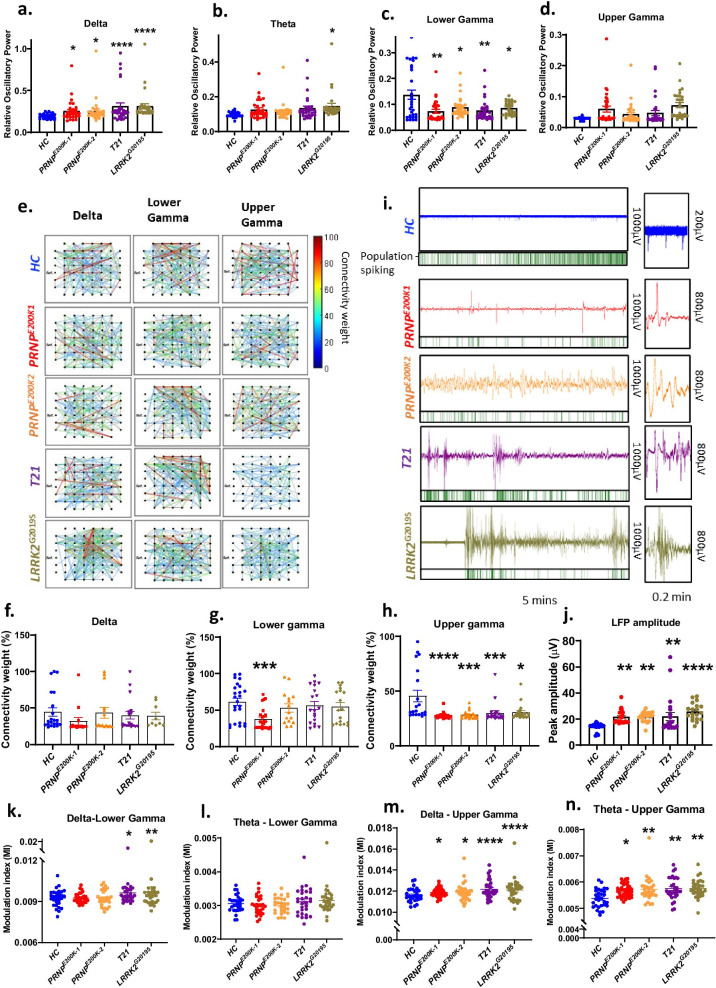
Genetic defects altered the neuronal oscillations. **a**–**d** Relative oscillatory power of delta, theta, low gamma, and upper gamma oscillations in 6–10 month- old *HC* (n = 30*)* and dCOs (*PRNP*^*E200K1*^, *PRNP*^*E200K2*^*, T21*, and *LRRK2*^G2019S^; n = 29). **e** Representative connectivity graphs of electrodes based on the inter-electrode correlation of delta, lower gamma, and upper gamma oscillatory power. **f**–**h** The weight (based on Pearson’s correlation coefficient) of the connectivity in **e** (n = 9 to 25). **i** Representative traces of local field potential (LFP) generated by each organoid line. The right panel is a magnification of the left panel. The bottom panel is a raster plot showing the spikes detected in the corresponding LFP. **j** The peak amplitudes of LFP. **k**–**n** The modulation index of the coupling of delta and theta phases with the amplitudes of the lower gamma oscillations (**k,** **l**; n = 30) and amplitudes of upper gamma oscillations (**m,** **n**; n = 30). **a**–**d f**–**h, j**–**n** Measurements were compared to the *HC* by One-way ANOVA on ranks with Dunnett’s correction for multiple comparisons. Each point on the graphs represents an individual organoid. Bars and error denote mean and SEM. * p < 0.05, **p < 0.01, ***p < 0.001, ****p < 0.0001

We also explored the frequency domain of the organoid neuronal activity to check for features known in electroencephalogram studies as physiological correlates of abnormal brain activity in humans. We focused on the 6–10 month-old COs because the changes we observed in the neuronal firing and communication in the dCOs relative to the *HC* were mainly in this age group (3–4 month-old CO data can be found in Additional file 12a*,* c). All the dCOs exhibited significantly enhanced delta oscillatory power, which are the slowest brain waves (Fig. 2a). The *LRRK2*^G2019S^ also showed enhanced oscillatory powers of theta and beta oscillations (Fig. 2b; Additional file 12f). The lower gamma oscillatory power was significantly reduced in all the dCOs (Fig. 2c). This inversely proportional relationship to the enhanced delta power implied slowing neuronal oscillations in these COs. The power of the upper gamma was not different in the dCOs (Fig. 2d). However, we observed significantly reduced strength of connected channels when measured by Pearson’s correlation coefficient (weight of connectivity) of upper gamma oscillatory power (Fig. 2e, h). The *PRNP*^*E200K*1^ also displayed weaker connectivity in the lower gamma oscillation (Fig. 2 g). Normal connectivity was observed in oscillations slower than gamma (Fig. 2f; Additional file 12g–i). The LFP amplitudes were significantly higher in all the dCOs (Fig. 2j), which correlated with the enhanced modulation of the amplitudes of upper gamma oscillations by delta and theta phases (Fig. 2m, n), an indication of strong coupling between these slow phases and upper gamma oscillations. Such increased coupling was not evident between other slower waves (alpha, beta, and lower gamma) and upper gamma oscillations (Additional file 12d). Only the *T21* and *LRRK2*^*G2019S*^ showed significantly enhanced coupling between the delta phase and lower gamma oscillations (Fig. 2k). All the dCOs showed no enhanced coupling between theta and lower gamma oscillations (Fig. 2l). These findings of increased coupling between slow and fast oscillations were consistent with periods of hyperactivity in the dCOs. We describe these activities as hypersynchronous events like spike-and-wave discharges, demonstrating a compromised excitatory-to-inhibitory balance in the dCOs. (Fig. 2j). These events appeared to reduce the spike rate in the dCOs relative to the *HC* (Fig. 2j). Overall, the abnormal events in the dCOs are associated with slowing neural oscillation and imbalanced excitatory-to-inhibitory neuronal functions [2, 31].

### dCOs demonstrate altered levels and activity of synaptic protein markers

**Fig. 3:**
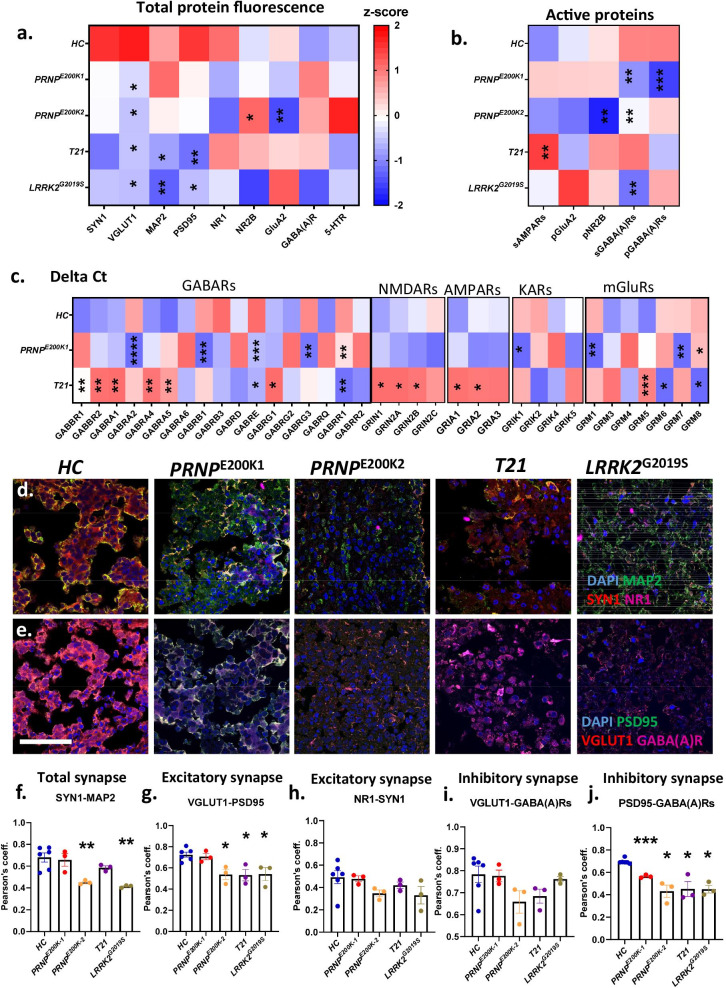
Genetic defects altered the expression levels and functions of synaptic markers that are essential for synapse formation. **a** A heatmap displaying the Z-scores of the total protein levels of essential synaptic proteins in 6–10-month-old *HC* and dCOs (*PRNP*^*E200K1*^, *PRNP*^*E200K2*^*, T21*, and *LRRK2*^G2019S^). See Additional file 13 for the representative images and quantifications. **b** A heatmap displaying the Z-scores of the active levels of essential synaptic proteins in *HC* and genetic defect organoids. See Additional file 14 for the representative images and quantifications. **a**, **b** Protein levels in the mutants were compared with the *HC* by One-way ANOVA with Dunnett’s correction multiple comparisons. **c** A heatmap displaying the Z-scores of the Delta Ct from the qRT-PCR analysis of various neurotransmitter receptors of *HC*, *PRNP*^*E200K1*^ and *T21* organoids. The mRNA levels in the organoids with genetic defects were compared to the *HC* by Two-way ANOVA with Dunnett’s correction for multiple comparisons. See Additional file 15 for additional data. **d** Representative confocal immunofluorescence images displaying the colocalizations of SYN1(red), MAP2(green), and NR1 (magenta). **e** Representative immunofluorescence images showing the colocalizations of VGLUT1 (red), PSD95 (green), and GABA(A)Rs (magenta). **f–j** The degree of colocalization between SYN1 and MAP2 (**f**), VGLUT1 and PSD95 (**g**), NR1 and SYN1 (**h**), VGLUT1 and GABA(A)Rs (**i**), and PSD95 and GABA(A)Rs (**j**). **f–j** n = 6 in *HC* and n = 3 in the genetic defect organoids. The average Pearson’s correlation coefficient or degree of colocalization was compared between organoid lines by One-way ANOVA with Dunnett’s correction for multiple comparisons. **a**–**c** The heatmap key is on the right panel of (**a**). **d** scale bar represents 50 μm. Each point on the graphs represents an individual organoid. Bars and error denote mean and SEM. * p < 0.05, **p < 0.01, ***p < 0.001, ****p < 0.0001

To further investigate the molecular mechanisms underlying the altered neuronal communication in the mature dCOs, we measured the levels of specific neuronal proteins essential for neuronal firing and synapse formation by immunofluorescence. The levels of total pre-synapse, as shown by the protein levels of synapsin1 (SYN1), were not altered in the dCOs; however, the levels of glutamatergic pre-synapse, as measured by the presence of a glutamate transporter VGLUT1, were significantly reduced in these COs (Fig. 3a; Additional file 13). MAP2 and PSD95, markers of dendritic spines and post-synaptic terminals [32], were significantly reduced in the *T21* and *LRRK2*^*G2019S*^, but normal in the *PRNP*^*E200K*^ COs (Fig. 3a; Additional file 13). This demonstrated a loss of dendritic spines and post-synaptic terminals in *T21* and *LRRK2*^*G2019S*^ that was not seen in the *PRNP*^*E200K*^ COs. The *PRNP*^*E200K2*^ uniquely showed increased total NR2B-containing NMDARs and reduced protein levels of GluA2-containing AMPARs (Fig. 3a; Additional file 13), molecular changes associated with long-term depression [32].

Next, we used LiveReceptor probes, which are fluorescently labelled ligands that indicate the availability of functional binding pockets, and antibody detection of phosphorylated (active receptors) to investigate the activity of receptor families (Fig. 3b; Additional file 14). This showed decreased GABA(A)R availability in all the dCOs except the *T21*. Despite its increased protein detection, the fraction of NR2B that was active was significantly reduced in the *PRNP*^*E200K2*^, suggesting that the increased detection of total NR2B was likely a compensatory attempt to improve its reduced activity (Fig. 3a*,* b; Additional files 13, 14). The levels of surface AMPARs, indicating the total active AMPARs, were normal in the dCOs except the *T21,* which showed significantly increased surface AMPARs. We also compared these with the mRNA expression of neurotransmitter receptors that are predominantly expressed on post-synaptic terminals including NMDARs, AMPARs, KARs, and GABARs in the *PRNP*^*E200K1*^ and *T21* organoids. We could not obtain mRNA from *PRNP*^*E200K2*^ and *LRRK2*^*G2019S*^ for RT-PCR analyses due to technical difficulty. The *T21*, but not the *PRNP*^*E200K1*^, expressed significantly more transcripts of the genes encoding the NMDAR subunits GRIN1, GRIN2A, and GRIN2B (Fig. 3C; Additional file 15). We also observed altered gene expression of other neurotransmitter receptors, which are important in neurophysiology and implicated in disease, including the metabotropic receptors (mGluRs), serotonin receptors (5HTRs), and cannabinoid receptors (Additional file 15). Of the post-synaptic markers examined, the GABAR family showed some of the most overt changes. The *T21* COs displayed an increase in the transcripts of five of the genes encoding the subunits of GABARs and reduced expression of two genes (Fig. 3C; Additional file 15). The *PRNP*^*E200K1*^ also significantly decreased the expression of five genes encoding the subunits of GABARs (Fig. 3C; Additional file 15). Together, we found that the expression levels of a broad spectrum of important neuronal markers are altered by the genetic defects, although the changes differ between disorders.

### Synapse number and composition is altered in dCOs

To look at the influence of the mutations on the synapse, we examined the major types of synapse including the total synapse, excitatory synapse, inhibitory synapse by confocal immunofluorescence. We measured the total synapse by the colocalization between SYN1 and MAP2 (Fig. 3d), the excitatory synapse by the colocalization between VGLUT1 and PSD95 and also NR1 and MAP2(Fig. 3e), the inhibitory GABAergic synapse by the colocalization between VGLUT1 and GABA(A)Rs as well as PSD95 and GABA(A)Rs (Fig. 3e). The degree of the colocalization was measured as Pearson’s correlation coefficient. Relative to the *HC, PRNP*^*E200K2*^ and *LRRK2*^*G2019S*^ exhibited low levels of total synapses (Fig. 3d*,* f). Excitatory synapses were significantly reduced in the *PRNP*^E200K2^, *T21*, and *LRRK2*^*G2019S*^, but not the *PRNP*^E200K1^ (Fig. 3e*,* g). However, excitatory synapses measured by the levels of NR1-containing NMDARs localized on the dendritic spines were not statistically different from the *HC* (Fig. 3d*,* h)*.* The numbers of GABAergic synapses were not statistically different from the *HC* when measured by the pairing of VGLUT1 and GABA(A)Rs (Fig. 3e*,* i). However, the colocalization between GABA(A)Rs and PSD95 was significantly lower in the dCOs than the *HC*, demonstrating reduced GABARs localized on the post-synapse, or the intra-synaptic GABARs (Fig. 3e*,* j). Thus, suggested substantial GABARs localized on either the pre-synapse or the extra-synapse and peri-synapse. The most effective molecular mechanism to change the efficacy of neuronal network communication is to alter the quantity and properties of synapses; this appears to occur due to the presence of the mutations.

### Neurotransmitter production and release are altered in the dCOs

**Fig. 4:**
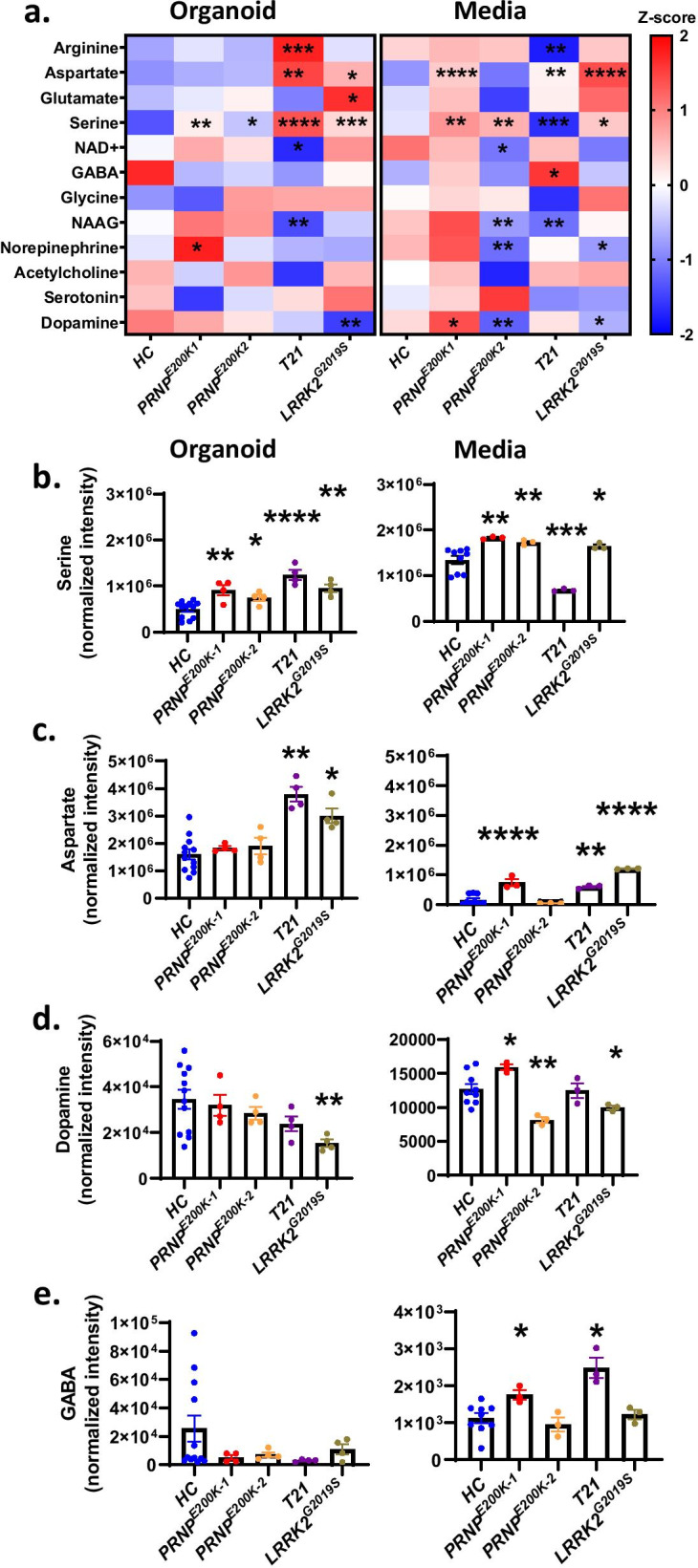
The genetic defects altered the production and release of neurotransmitters. **a** Heatmaps displaying the Z-scores of the levels of neurotransmitters, extracted from organoids and media, in *HC and* dCOs (*PRNP*^*E200K1*^, *PRNP*^*E200K2*^*, T21*, *LRRK2*^G2019S^). The heatmap key is on the right panel. **b**–**e** Some of the raw data summarized in **a** including serine (**b**), aspartate (**c**), dopamine (**d**), and GABA (**e**). **b–e** consisted of n = 12 for the *HC* and n = 4 for the other organoid lines. Levels of neurotransmitters in the organoids with genetic defects were compared to the *HC* by One-way ANOVA with Dunnett’s correction for multiple comparisons. Each point on the graphs represents an individual organoid. Bars and error denote mean and SEM. * p < 0.05, **p < 0.01, ***p < 0.001, ****p < 0.0001

With synapse composition changed in the dCOs, we looked at their neurotransmitter complement. We used metabolomics to measure the levels of various neurotransmitters extracted from the organoids to estimate their ability to produce neurotransmitters and the levels of neurotransmitters in the media to estimate the levels of release (indicative of the degree in which the organoids utilized neurotransmitters for neuronal electrical signaling). Six-month old COs appeared to release more neurotransmitters than 3-months old COs, an indicator of increased usage of neurotransmitters for electrical signaling at 6–10 months old (Additional file 16 a, b). Assessing production and release at 6–10 months old showed that dCOs appeared to produce and release excessive levels of serine and/or aspartate, which are excitatory neurotransmitters, and that the *T21* organoids showed the most changed profile in production and secretion (Fig. 4a–e). As expected, the *LRRK2*^G2019S^ COs produced very low dopamine and released low norepinephrine (Fig. 4a, d). The production of GABA was normal in the dCOs, but the release was increased in the *PRNP*^*E200K1*^ and *T21*. The normal production of GABA was consistent with the levels of parvalbumin-positive neurons, the primary marker of GABAergic interneurons that produce GABA (Additional file 16c). The increased synthesis and release of excitatory neurotransmitters in the dCOs could be an attempt to increase the activity of NMDARs and rescue the neuronal excitability and network communication.

### DCOs show altered electrophysiology following stimulation of ionotropic receptors

**Fig. 5:**
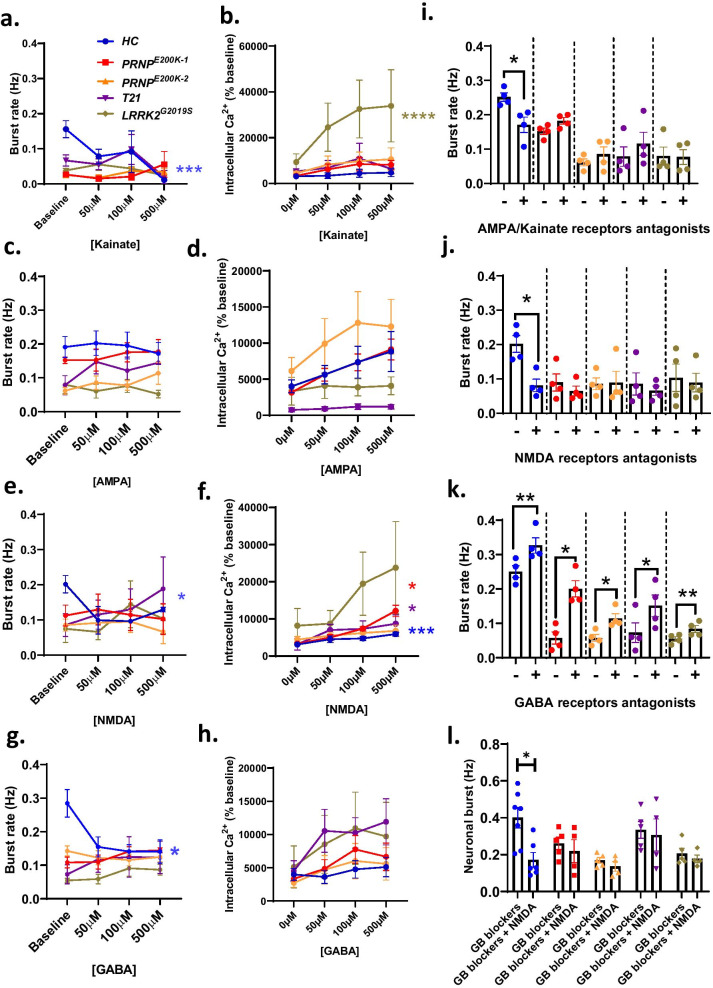
The genetic defects altered the agonist-dependent activity of the major ionotropic receptors. **a**, **c**, **e**, **g** Burst rate recorded in 6–10-month-old *HC* and dCOs (*PRNP*^*E200K1*^, *PRNP*^*E200K2*^*, T21*, and *LRRK2*^G2019S^) before and after exposure to increasing concentrations of kainate (**a**; n = 4), AMPA (**c**; n = 4), NMDA (**e**; with 5 µM of glycine; n = 4), and GABA (**g**; n = 4). **b**, **d**, **f**, **h** Intracellular levels of calcium before and after exposure to increasing concentrations of kainate (**b**; n = 4-), AMPA (**d**; n = 4–7), NMDA (**f**; with 5 µM of glycine; n = 4–7), and GABA (**h**; n = 4). **i–k** Burst rate before and after treatments with 30 μM NBQX (**i**; n = 4), 100 μM AP5 and 10 µM maleate solution (**j**; n = 4), and 100 µm Bicuculine and 10 μM CGP55845 hydrochloride (**k**; n = 4). **l** Burst rate before and after stimulating the organoids with 500 μM NMDA/5 µM glycine in the presence of GABAR (GB) receptors blockers, 100 µM Bicuculine and 10 μM CGP55845 hydrochloride (n = 7 for the *HC*; n = 4 for other organoid lines). The dose- response (mean burst rate or intracellular calcium) to each treatment was compared between organoids by Repeated Measures Two-way ANOVA with Dunnett’s correction for multiple comparisons. **i–l** We used paired Student’s t-test to compare the average burst rate before and after treatments. Each point on the graphs represents an individual organoid. If not otherwise indicated CO colour code are as follows; *HC* (blue), *PRNP*^*E200K−1*^ (red), *PRNP*^*E200K−2*^ (yellow), *T21* (purple) and *LRRK2*^*G2019S*^ (grey). Bars and error denote mean and SEM. * p < 0.05, **p < 0.01, ***p < 0.001, ****p < 0.0001. **a**–**h** Asterisk colour signifies which organoid line with the statistically significant results

Given the vital roles of ionotropic receptors in maintaining neuronal network connectivity, we next considered if the mutations altered the normal roles of these receptors, thereby affecting neuronal communication. We exposed the organoids to increasing doses (50, 100, and 500 µM) of agonists of the major ionotropic receptors including the KARs, AMPARs, NMDARs, and GABARs while recording neuronal communication. We also measured the intracellular calcium following the chemical stimulations. There is a direct correlation between the activity of the ionotropic receptors and the levels of intracellular calcium, and a direct link between high intracellular calcium and excitotoxicity [33]. Kainate, NMDA (with glycine as a co-agonist) and GABA treatments significantly reduced the burst rate in the *HC* but the dCOs, with their lower baseline burst rate, were unaffected (Fig. 5a, e, g). Despite the lack of burst rate change in the dCOs, some increases in calcium flux were observed, most notably in response to NMDA, indicating that NMDARs were hyperactivated. The AMPA treatment did not significantly change the burst rate or calcium flux of all the COs relative to the baseline (Fig. 5c, d). Altogether, the genetic mutations appeared to alter the activity of the excitatory ionotropic receptors associated with normal neuronal communication while increasing the calcium influx into the cells, possibly through a mechanism whereby the activity of the ionotropic receptors is unrelated to neuronal communication.

To determine whether the lack of response of the dCOs to agonists of the major ionotropic receptors was due to these receptors being dysfunctional, we treated the organoids with antagonists of these receptors. NBQX, a blocker of AMPARs and KARs, significantly reduced the burst rate in the *HC*, but not in the dCOS (Fig. 5i). Similar results were observed when the NMDARs were blocked by a cocktail of AP5 and maleate (Fig. 5j). A cocktail of Bicuculine and CGP, antagonists of GABA(A) and GABA(B) receptors, significantly increased the neuronal burst rate of all COs, albeit the dCOs were lower and did not reach the activity of the *HC* COs (Fig. 5k). Hyperactivation of NMDARs in the absence of GABAergic inhibition reduced *HC* neuronal bursts but exerted no effect on the dCOs (Fig. 5l). Collectively this indicated that both the excitatory and inhibitory functions of neurons were compromised.

### The GABAR—mediated function of NKCC1 is altered in the *T21* only

**Fig. 6:**
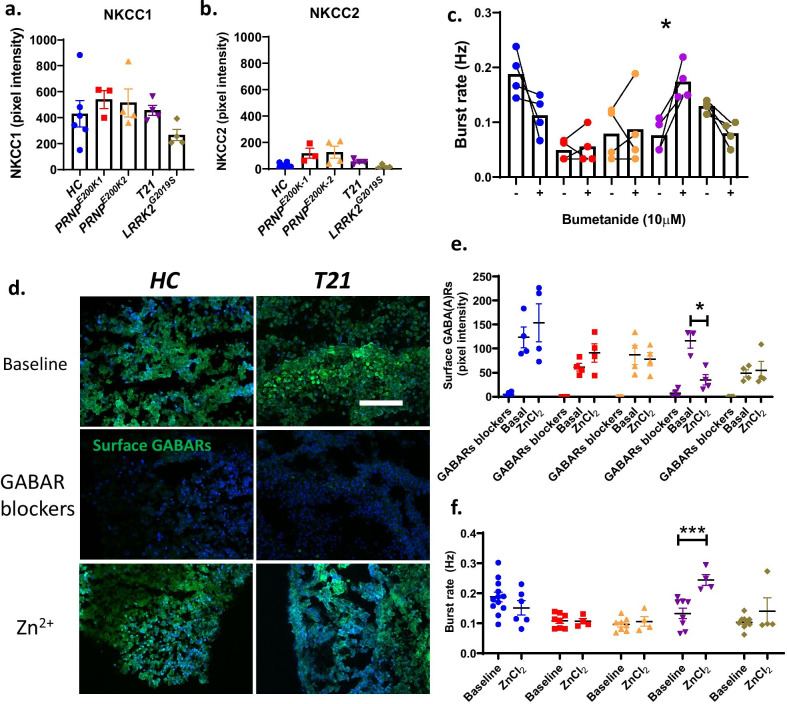
*T21* impaired NKCC1 activity and increased the activity of extra-synaptic GABARs. **a,** **b** Protein levels of NKCC1 and NKCC2 in 6–10-month-old *HC* (n = 6) and dCOs (*PRNP*^*E200K1*^ with n = 3, *PRNP*^*E200K2*^with n = 5*, T21* with n = 4, and *LRRK2*^G2019S^ with n = 4). Representative images are in Additional file 17. **c** Burst rate in response to a treatment with 10 µM NKCC1 blocker bumetanide (n = 4 per an organoid line). **d** Representative images of surface GABARs (green) before and after treatments with GABAR blockers (100 µM Bicuculine and 10 μM CGP55845 hydrochloride) or 500 μM ZnCl_2_ in *HC* and *T21* organoids. Scale bar is 100 µm. **e** Quantifications of the levels of surface GABARs before and after treatments with either GABARs blockers or ZnCl_2_ (n = 4). **f** Burst rate before (n = 24 for the *HC* and n = 16 for the other organoid lines) and after exposure to 500 μM ZnCl2 (n = 6 for the *HC* and n = 4 for the other organoid lines). **c**–**f** Average burst rates and levels of surface GABARs before and after the treatments with Bumetanide or ZnCl2 were compared by paired Student’s t-test. Each point on the graphs represents an individual organoid. If not otherwise indicated CO colour code are as follows; *HC* (blue), *PRNP*^*E200K−1*^ (red), *PRNP*^*E200K−2*^ (yellow), *T21* (purple) and *LRRK2*^*G2019S*^ (grey). Bars and error denote mean and SEM. * p < 0.05, **p < 0.01, ***p < 0.001

Given the evidence so far of impaired GABAergic inhibition, we explored various pathways known to modulate this inhibition to provide some mechanistic insights into the observed neuronal dysfunction. One of the factors modulating the activity of GABAergic neurons is the chloride homeostasis that is regulated by Na–K–Cl cotransporters (NKCCs) [13]. NKCCs exist in two types, NKCC1 and NKCC2. NKCC1 transports Cl^−^ into the cells and NKCC2 removes Cl^−^ out of the cells, thereby controlling the intracellular Cl^−^ gradient and the activity of GABARs [13]. We found no difference in the levels of NKCCs in the dCOs relative to the *HC* (Fig. 6a*,* b; Additional file 17). Blocking of NKCC1 activity with 10 µM bumetanide significantly increased the neuronal burst rate in the *T21*, but not in the other dCOs and *HC* (Fig. 6c). This finding suggested that the *T21* appeared to increase the Cl^−^ influx via the NKCC1 and corrupted the inhibitory function of the GABARs. Thus, the *T21*, but not the other dCOs, have advanced dysfunction of GABAergic neuronal activity due to a disrupted role of NKCC1 in the homeostasis of Cl^−^.

### The activity of extra-synaptic GABARs is excessive in *T21* only

One factor that modulates the activity of GABARs is their localization. GABARs that are on the synapse produce phasic or rapid inhibition, whereas those on the peri-synapse and extra-synapse induce tonic or prolonged inhibition. Based on our observations in Fig. 3, the reduced levels of intra-synaptic GABA(A)Rs in the dCOs were inconsistent with the unaltered total levels of GABA(A)Rs, thus suggesting possible high levels of extra-synaptic GABARs in these organoids. We next looked at whether the GABARs were hyperactive on the extra-synapse and peri-synapse compared to the synapse in the dCOs. We labelled the surface GABARs with a LiveReceptor probe against GABA(A)Rs before and after exposure to GABAR inhibitors (Bicuculine and CGP), or Zn^2+^ (ZnCl_2_), a negative modulator of extra-synaptic GABARs (although it plays many other roles in neurons [34, 35]). The GABAR inhibitors abolished the surface GABARs in all organoids (Fig. 6e). The Zn^2+^ did not change the levels of surface GABARs in the *HC* and most of the dCOs, except for the *T21*, where the surface GABARs were partially but significantly reduced (Fig. 6d, e). Only the *T21* showed a significant increase in neuronal burst rate after exposure to Zn^2+^ (Fig. 6f), but not the *HC* and the other dCOs. We also found that Zn^2+^ did not increase the activation of caspase-3 in all organoid lines, demonstrating no Zn^2+^ related toxicity involved (Additional file 18). Overall, only the *T21* showed possible abnormal hyperactivity of the extra-synaptic GABARs.

### The dCOs demonstrate altered neurosteroid dependent modulation of GABARs activity

**Fig. 7:**
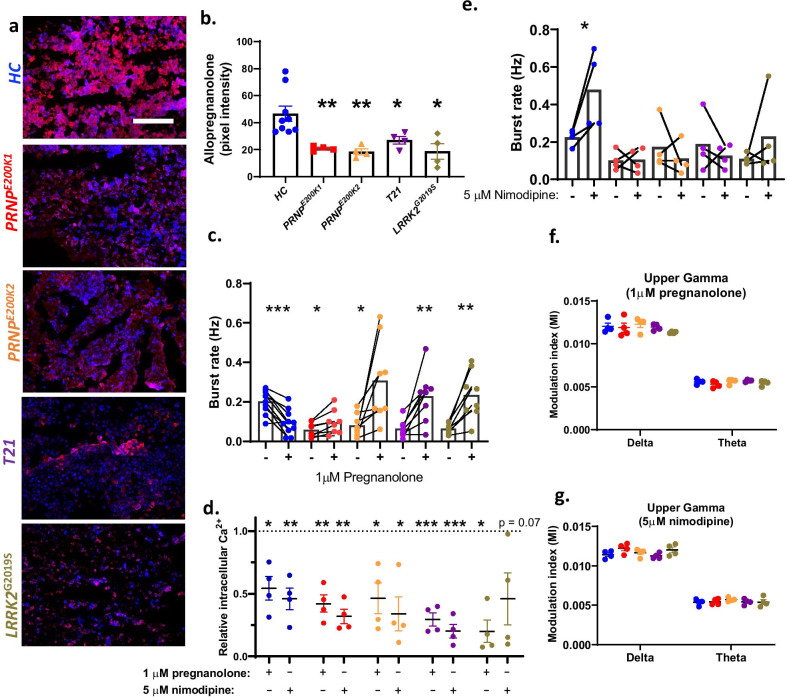
The genetic defects altered neuronal communication associated with neurosteroid Allopregnanolone. **a,** **b** Representative images and quantifications of neurosteroid allopregnanolone detected in 6–10-month-old *HC* (n = 9) and dCOs (*PRNP*^*E200K1*^, *PRNP*^*E200K2*^, *T21*, *LRRK2*^G2019S^; n = 4). Scale bar is 100 µm. **c** Burst rate before and after treatments with 1 µM pregnanolone (n = 10 for the *HC*; n = 8 for the other organoid lines). **d** Relative intracellular calcium levels measured before and after treatments with either 1 µM pregnanolone or 5 μM nimodipine (n = 4 per an organoid line). **e** Burst rate before and after the treatments with 5 µM nimodipine (n = 4 per an organoid line). **f, g** The modulation index of the coupling between the delta/theta phases and the amplitudes of the upper gamma oscillations after treatments with pregnanolone (**f**; n = 4) and nimodipine (**g**; n = 4). **b** Allopregnanolone levels were compared between groups by One-way ANOVA with Dunnett’s correction for multiple comparisons. **c,** **e** Effect of the treatments within an organoid line was analysed by Paired Student’s t test. **d** Calcium levels after treatments were normalized to the pre-treatment levels and the effect of the treatments on the calcium flux was determined by One-sample t-test based on a hypothetical value of 1. **f,** **g** Measurements were analysed by Two-way ANOVA with Dunnett’s correction for multiple comparisons. Each point on the graphs represents an individual organoid. If not otherwise indicated CO colour code are as follows; *HC* (blue), *PRNP*^*E200K−1*^ (red), *PRNP*^*E200K−2*^ (yellow), *T21* (purple) and *LRRK2*^*G2019S*^ (grey). Bars and error denote mean and SEM. * p < 0.05, **p < 0.01, ***p < 0.001

GABARs are also modulated by neurosteroids. One such neurosteroid is allopregnanolone, which is known to facilitate the activity of GABARs. Allopregnanolone is reduced in a spectrum of psychiatric and neurological disorders including AD and depression and anxiety [36, 37]. The levels of allopregnanolone were significantly reduced in all the dCOs relative to the *HC* (Fig. 7a, b). We then measured the neuronal response to a brief exposure to 0.5 μM and 1 µM pregnanolone, a synthetic equivalence of allopregnanolone. The 1 µM pregnenolone significantly inhibited the neuronal burst in the *HC*, while significantly increasing the neuronal burst rate in all the dCOs (Fig. 7c). The 0.5 µM pregnanolone significantly increased the neuronal burst rate in only the *PRNP*^*E200K2*^, *T21*, and *LRRK2*^*G2019S*^ (Additional file 19)*.* Further, pregnanolone is also known to inhibit glutamate release and activities of various calcium channels including the L-type calcium channels [38]. To determine if the improvement in the neuronal communication of the dCOs was linked to reduced calcium influx, we measured the intracellular calcium levels before and after exposure to pregnanolone and nimodipine (a potent-calcium channel blocker). We also measured the neuronal communication following the exposure to nimodipine. Both pregnanolone and nimodipine reduced the calcium flux in all organoids (Fig. 7d). Interestingly, nimodipine significantly increased the burst rate of the *HC*, but not the dCOs (Fig. 7e). This result revealed that the levels of intracellular calcium are not directly linked to neuronal communication deficits observed in the dCOs. Further, the modulation of the amplitude of the upper gamma oscillations was no longer different to the *HC* after the treatments with pregnanolone and nimodipine (Fig. 7f*,* g). Overall, neuronal communication in the dCOs could be improved by using pregnanolone to positively modulate the activity of GABARs.

## Discussion

Herein we used cerebral organoids generated from donors carrying the genetic mutations, *PRNP*^*E200K*^, *T21*, and *LRRK2*^G2019S^, which are directly linked to the neurological diseases genetic CJD, DS, and PD respectively, to determine the effect on neuronal network communication. We found that all the organoids were able to generate mature neuronal activity at 6–10 months old, revealing that the mutations did not significantly hinder the processes of neuronal maturation. However, electrophysiological differences were observed including reduced neuronal network communication, slowing neuronal oscillations, and increased modulations of gamma band amplitudes by delta and theta phases. The latter were associated with the detection of hyperactive events like spike-and-wave discharges. These altered neuronal dysfunctions were linked with changed neurotransmitter receptor expression and neurotransmitter production and release. Further changes included reduced levels of synapses including the GABAergic synapses, which correlated with the compromised functions of the major excitatory ionotropic receptors such as the kainate, NMDA, and GABA receptors. The disrupted functions were associated with the reduced levels of neurosteroid allopregnanolone and its role as a positive modulator of GABARs. Further, advanced GABAergic neuronal dysfunctions, linked to impaired activity of NKCC1 and hyperactivity of extra-synaptic GABARs, were observed in the *T21*, the only mutation here known to manifest mental deficit at birth. Together our findings indicate that, while these dCOs may not be manifesting a disease phenotype, their neurophysiology is altered.

Our cerebral organoid mutation models exhibited slow neural oscillation and altered neuronal network communication without evidence of disease-related pathology. Neuronal dysfunction is often evident during clinical disease. However, previous studies have shown slowing of the cortical oscillation [39] and cortical hyperintensity on diffusion-weighted MRI in carriers of *PRNP* E200K mutation prior to clinical disease [40, 41]. Further, neuronal oscillations are altered in mouse models of AD prior to the deposition of amyloid beta [42], and in individuals with *T21* prior to the onset of AD [43]. The brain functional connectivity is reduced in asymptomatic carriers of LRRK2 mutations and progressively becomes exacerbated at the clinical onset of PD [44, 45]. Slowing neuronal oscillation is associated with seizure or epileptiform like events [46, 47]. Collectively this indicates the presence of the mutations is sufficient to induce significant functional changes within neurons.

The organoids with genetic mutations exhibited hypersynchronous events like patterns associated with non-convulsive seizure. These events were associated with the strong peak amplitude of the high frequency neuronal firing and the enhanced modulation of the amplitudes of the gamma oscillations by the delta and theta phases in these organoids. Neuronal events associated with non-convulsive seizure or absence seizure are observed in animal models and patients of numerous mental illnesses including anxiety and depression, schizophrenia, and bipolar disorder [48–53]. This suggests that such abnormal events arise largely from abnormal neuronal functions rather than degeneration of neurons. Non-convulsive seizure is one of the most common types of seizure in CJD and it appears to be more frequent in genetic CJD [54–56]. An animal model of genetic CJD has demonstrated slow waves and sharp spikes with increased susceptibility to kainite-induced seizure [57, 58]. Further, seizure has been observed in animal models of AD [59, 60] and in AD patients at a very early stage of dementia [55, 61], and also in 84% of DS individuals who developed dementia and AD [62, 63]. PD is associated with epileptic seizure [56], whereby abnormally reduced levels of dopamine is linked to absence seizure [64]. These functional aberrations appear to occur in genetic disease because of the mutation, and it is likely that they become exacerbated around the time of symptomatic disease onset.

Spike-and-wave events are linked to imbalanced excitatory to inhibitory activity, a physiological deficit known to correlate with dysfunctional GABAergic inhibitory system [65]. We observed impaired activity of GABAergic neurons in the dCOs. Our data demonstrated a diminished level of synaptic GABARs in these organoids, suggesting a reduced degree of rapid inhibition and a potential mechanism for the spike-and-wave events we observed here [66]. The reduced activity of GABARs was evident in low levels of surface GABARs in all the dCOs except the *T21*, which seemed to express dysfunctional extra-synaptic GABARs. Abnormal activity of NKCC1 exacerbated the disrupted role of GABARs in *T21* COs, an impairment that was reversed by NKCC1 blocker bumetanide. Similarly, in a mouse model of DS, the abnormal activity NKCC1 triggered GABARs to excite neurons instead of cause inhibition, which was rescued by blocking NKCC1 [14]. The production and release of GABA were largely normal in all organoids, despite the increased release in the *PRNP*^*E200K1*^ and *T21,* suggesting that if the available GABARs were enough to regulate proper neuronal excitability, then maximizing their activity could correct the unbalanced excitatory-to-inhibitory activity. This was achieved by addition of a neurosteroid, pregnanolone, which is a positive modulator of GABARs and for which endogenous levels were deficient in all the dCOs. Importantly, this neurosteroid is deficient in numerous neurological diseases and mental disorders including AD and depressed mood [67–69] and has been shown to increase lifespan and delay onset of symptoms in a Neiman-Pick disease mouse model [70].

Previous studies have found that cerebral organoids generated from DS iPSCs produce an over-abundance of specific subclasses of GABAergic interneurons as part of a neurodevelopmental abnormality linked with increased expression of OLIG2 [71]. Another study, using iPSCs to differentiate GABAergic interneurons directly found the neurons developed less complexity, altered subtype preference and reduced migration capacity [72]. We did not see a difference in the staining of interneurons, instead observing a small decrease in inhibitory synapses with GABARs and dysfunctional extra-synaptic GABARs. Our results differ from the earlier organoid study likely because we utilized organoids much later in development (3 months or older as opposed to 5–8 weeks in vitro [71]) and the authors saw the most convincing changes at 5 weeks post differentiation. This may indicate that an initial burst of interneuron differentiation is not sustained, and the population functionally declines as organoids age. While we did not consider the many possible changes that could be occurring within the composition of the interneuron population, and this may be critical to investigate in the future, the dysfunctional extra-synaptic GABARs we observed within the GABAergic system do further support failures previously reported.

That the GABAergic system is affected by all three mutations is interesting and consistent with current knowledge. GABAergic dysfunction has been linked with the developmental cognitive impairments in DS [73, 74] and GABAergic collapse has been implicated in deterioration during PD, corresponding with Braak stage, and as a potential therapeutic target [75, 76]. In post-mortem brain tissue from CJD patients parvalbumin-immunoreactive interneurons are lost and this appears to begin with destruction of the surrounding extra-cellular matrix [77–79]. Similar results have been observed in animal models of PrD [80–83]. In addition, some cases of CJD with non-convulsive status epilepticus are responsive to benzodiazepines, positive modulators of GABA receptors [84, 85]. While the organoids examined herein are not manifesting disease markers, the influence of the mutation on GABAergic neurotransmission supports that these neurons are dysfunctional and may be vulnerable to loss during disease.

That the GABAergic system is implicated in each of these diseases and that the organoids replicated this dysfunction will allow us to probe more deeply into such failures with the human brain. For example, despite the consistent neurophysiological alteration in all the dCOs, the underlying molecular mechanisms appeared to be quite variable. This observation aligns with the fact that while there are overlaps in the clinical presentations of the E200K CJD, *LRRK2*^G2019S^ and DS-causing AD such as depression and dementia, the disease-causing mutations and the corresponding pathology are different. The genetic background variability of the dCOs might be linked to the inconsistency of the molecular mechanisms underlying the change in their neurophysiology. Despite carrying the same disease-causing mutation, the two lines of *PRNP*^*E200K*^ organoids, from two different donors, expressed some of the key synaptic receptors, formed synapses, and produced and secreted neurotransmitters differently. This observation is consistent with the different penetrance of CJD and age of clinical onset in carriers of *PRNP*^*E200K*^ mutation [86, 87] and indicates the importance of factors beyond the single mutation in determining the functional neuronal phenotype.

Although the CO model offers a remarkable insight into the changes that occur in three-dimensional human brain, the COs have limitations [88, 89]. Mature cerebral organoids have been reported to exhibit neurophysiological activity equivalent to the cerebral cortex of a neonate [65], and therefore, the age-dependent growth we observed was limited to an early stage in neonatal growth. The average age of clinical onset in the PRNP^E200K^ CJD, genetic *LRRK2*^G2019S^ PD, and DS causing AD falls within the adulthood age-range, thus supporting that the organoids were less likely to exhibit pathology related to clinical diseases. However, DS organoids have been shown to develop AD-like pathology [6]. One possible explanation for our contradictory finding is that our donor is one of the reported ~ 50% DS individuals that do not develop AD-like pathology [90]. In addition, we observed no PD-related pathology in the *LRRK2*^G2019S^ organoids, although the donor was already symptomatic (with 2 year history of PD and depression) at the time of the fibroblast collection. This latter deviation from previous literature reports has most likely arisen from the different differentiation protocols applied. We used non-directed differentiation into cerebral organoids, whereas studies specifically targeting PD have generated mid-brain organoids that more closely represent the region that is damaged during PD [7, 91]. Therefore, it is probable that the cerebral organoids, representing a region that is less affected during disease, did not show similar pathology in our system to that observed in the midbrain organoids. Furthermore, generating organoids by non-guided methods, including the Lancaster & Knoblich protocol used here [88], produces organoids with a greater diversity of cell types than systems that direct differentiation down defined pathways [89], therefore a level of batch-to-batch variability may have contributed variability into the recorded results. Additionally, given that organoid technology is under constant development to better the experimental model, we acknowledge that the current limitations of this technology may necessitate further experimentation to be undertaken for validation of some of the observed phenotypes. However, despite these limitations, the mutations have a sufficiently pronounced effect on cellular biology that we were able to discern neuronal dysfunction in the absence of traditional disease markers.

## Conclusions

Here we demonstrated impaired neuronal communication in cerebral organoids differentiated from fibroblasts donated by carriers of *PRNP*^*E200K*^, *T21*, and *LRRK2*^G2019S^ genetic mutations, associated with CJD, DS, and PD respectively. These organoids exhibited no clear evidence of disease-related pathology. The neuronal dysfunction was associated with the presence of neuronal events like spike-and-wave discharges, which were linked with abnormal GABAergic neuronal inhibition. The synaptic GABARs were reduced, demonstrating a possibility of poor phasic inhibition in these organoids. The dysfunction of GABARs in the *T21* was also mediated by an abnormal role of the chloride transporter NKCC1. The neuronal communication in these organoids were significantly improved by a treatment with neurosteroid pregnanolone, a positive modulator of GABARs. Altogether our results support that, in human cerebral organoids, the GABAergic system is impaired across several genetic conditions where cognitive symptoms are common, and that targeting this system may offer a future means of alleviating some of these symptoms.

## Supplementary Information

**Additional file 1:** Neuronal firing and network communication. **(a)** Neuronal burst rate before and after exposure to TTX. **(b)** The strength (weight) of the connectivity between electrodes based on spike correlation in the healthy control organoids (*HC*) and organoids with genetic mutations (*PRNP*^*E200K1*^, *PRNP*^*E200K2*^*, T21*, and *LRRK2*^G2019S^) at 3–4 months (n = 8–11) and 6–10 (n = 17–21) months old. **(c)** Intracellular levels of calcium in all organoid lines at 6–10 months old (n = 16 for the *HC* and n = 28 in the other lines). **(d, e)** Burst rate (d) and inter-spike interval coefficient of variation (CV; e) in each organoid line at 3–4, 6–7, and 8–10 months old. **(f)** Burst rate of the three *HC* lines at 6–10 months old. Each point on the graphs represents an individual organoid. Bars and error denote mean and SEM.

**Additional file 2:** This file contains codes used to extract the electrophysiology raw data for further analyses.

**Additional file 3:** This file contains codes used to calculate the connectivity based on the relative oscillatory power.

**Additional file 4:** This file contains codes used to calculate the phase-amplitude coupling.

**Additional file 5:** This file contains a function, [MID, ~ ,MIT, ~] = Mi(x), used in the Additional file 4 to calculate the modulation index (MI) of the delta (D) and theta (T) phases in the local field potential (x).

**Additional file 6:** This file contains a function, [MIA, ~ ,MIB, ~] = MiA(x), used in the Additional file 4 to calculate the modulation index (MI) of the alpha (A) and beta (B) phases in the local field potential (x).

**Additional file 7:** This file contains a function, [MIG, ~ ,MIGP, ~] = MiG(x), used in the Additional file 4 to calculate the modulation index (MI) of the lower gamma (G) and upper gamma (GP) phases in the local field potential (x).

**Additional file 8:** Primary antibody List.

**Additional file 9:** Spectral information for neurotransmitters characterized on Sciex5500 QTRAP® mass spectrometer using ESI ionization.

**Additional file 10:** MRM information for molecular species targeted. DP: declusteringpotential, EP: entrance potential, CE: collision energy, CXP: collision cell exit potential. All source conditions are in volts.

**Additional file 11:** Assessing for disease-related pathology. Representative images showing immunofluorescence detection of total amyloid beta (**a**; antibody: 6E10), alpha-synuclein (**b**; antibody: anti-alpha-synuclein), PrP (**c**; antibody: EP1802Y), and THT (**d**) in 6 month old healthy controls (*HC*) and organoids with genetic defects (*PRNP*^*E200K1*^, *PRNP*^*E200K2*^*,T21*, *LRRK2*^G2019S^) as indicated. Scale bar is 500 µm. Corresponding plots (right) show pixel intensity quantification in DAPI-positive cells. **(e)** Western blot analysis of APP/amyloid beta in lysates from all the organoids and a brain homogenate from a terminal Alzheimer’s disease (AD) brain (antibody: 6E10). The arrows depict bands corresponding to the molecular mobility of APP and Aβ42. The right panel shows the total protein loaded for each sample. **(f)** A western blot analysis of insoluble Tau (solubilized by sarkosyl and pelleted by ultracentrifugation) in all the organoid lines and a brain homogenate from a terminal Alzheimer’s disease (AD) brain (antibody: T22). The right panel shows the total protein loaded for each sample. Bars and error denote mean and SEM. The levels of amyloid beta, alpha-synuclein, PrP, and THT were compared between organoid lines by One-way ANOVA on ranks with Dunnett’s correction for multiple comparisons. **(g)** Primary ThT fluorescence data of K12 Tau RT-QuIC. Representative traces from *T21* tau-free mouse brain (KO), cerebrovascular disease (CVD), and Alzheimer disease (AD) brain homogenates, and Down syndrome (DS) organoid homogenates at 10^–3^ dilutions are displayed. Samples were also tested at 10^–3^-10^–6^ for CVD, 10^–3^-10^–10^ for AD, and 10^–2^-10^–5^ for DS. For the control brain homogenates, the curves are displayed as the average of 4 replicate wells. For the DS organoid homogenates, the curve is displayed as an average ± standard deviation of 5 independent organoids run in quadruplicate. **(h)** Primary ThT fluorescence data of α-synuclein RT-QuIC. Representative traces from Corticobasal degeneration (CBD) and Parkinson’s Disease (PD) brain homogenates, and organoid homogenates with (PD) or without (Control) the *LRRK2*^G2019S^ mutation at 10^–4^ dilutions are displayed. For the control brain homogenates, the curves are displayed as the average of 4 replicate wells. For the organoid homogenates, the curve is displayed as an average ± standard deviation from the indicated number of independent organoids run in quadruplicate. The dashed line indicates the ThT fluorescence threshold for a sample to be considered positive (see methods). (**i**) Immunofluorescence images of GFAP (active astrocytes) in 6-month-old HC and dCOs. The scale bar is 100 μm.

**Additional file 12:** Neural oscillations at 3–4 months. **(a)** Narrow band oscillatory power in 3–4-month-old healthy controls (*HC*) and organoids with genetic mutations (*PRNP*^*E200K1*^, *PRNP*^*E200K2*^*, T21*, and *LRRK2*^G2019S^; n = 18 to 24). **(b)** Local field potential (LFP) peak amplitudes in all organoid lines at 3 months old. **(c)** The modulation index of the coupling between theta phase and the amplitudes of the upper gamma oscillations at 3 months old. **(d)** The modulation index of the coupling between the phases of alpha, beta, and lower gamma and the amplitudes of upper gamma oscillations in 3-month-old organoids. **(e, f)** Relative oscillatory power of alpha and beta bands in 3-month-old organoids. **(g-i)** The strength (weight/Pearson’s coefficient) of the connectivity between electrodes based on the correlation of oscillatory power. **(a, d, e-i)** Analysed by Two-way ANOVA with Dunnett’s correction for multiple comparisons. **(b, c)** Analysed by One-way ANOVA with Dunnett’s correction for multiple comparisons. Each point on the graphs represents an individual organoid. Bars and error denote mean and SEM. * p < 0.05, **p < 0.01, ***p < 0.001, ****p < 0.0001.

**Additional file 13:** Total protein levels of key synaptic receptors. Representative immunofluorescence images showing detection of key synaptic proteins in 6–10-month-old healthy control organoids (*HC*) and organoids with genetic mutations (*PRNP*^*E200K1*^*, PRNP*^*E200K2*^, *T21*, and *LRRK2*^*G2019S*^) as indicated. Scale bar is 500 µm. The insets in the images of Syn1 and MAP2 show a high magnification images of these markers and their localization. Quantification of the pixel intensity of synaptic proteins (plots are shown right of the corresponding marker) in DAPI-positive cells was compared between organoid lines by One-way ANOVA on ranks with Dunnett’s correction for multiple comparisons. The quantification was done in the whole organoid section (imaged at 4 × magnification). Each point on the graphs represents an individual organoid. Bars and error denote mean and SEM* p < 0.05, **p < 0.01.

**Additional file 14:** Expression levels of active synaptic proteins. Representative immunofluorescence images showing detection of surface AMPA receptors (sAMPARs), phosphorylated GluA2-containing AMPARs (pGluA2), phosphorylated NR2B-containing NMDA receptors (pNR2B), surface GABA receptors (sGABARs), and phosphorylated GABA(A) receptors (pGABA(A)Rs) in 6–10-month-old healthy control organoids (*HC*) and organoids with genetic mutations (*PRNP*^*E200K1*^, *PRNP*^*E200K2*^*,T21*, and *LRRK2*^G2019S^) as indicated. Quantification of pixel intensity (in DAPI-positive cells) of the surface AMPARs and GABARs and ratios of phosphorylated (active) GluA2, NR2B and GABA(A). Active protein detection was compared between organoid lines by One-way ANOVA on ranks with Dunnett’s correction for multiple comparisons. Each point on the graphs represents an individual organoid. Bars and error denote mean and SEM. Scale bar is 500 µm. * p < 0.05, **p < 0.01, ***p < 0.001.

**Additional file 15:** Expression of genes for neurotransmitter receptors at 6 months old. **(a)** Pearson correlation heatmap and average linkage hierarchical cluster analysis of the row Z-scores of the Delta Ct from the qRT-PCR analysis of various neurotransmitter receptors in 6–10-month-old healthy control organoids (*HC*) and organoids with genetic mutations (*PRNP*^*E200K1*^ and *T21*). **(b-c)** Some of the Delta Ct data presented in **a**, displaying the mRNA levels for the subunits of GABA receptors (**b**), NMDA receptors (**c**), AMPA receptors (**d**), kainate receptors (**e**), and metabotropic receptors (**f**; n = 3 per an organoid line). Expression levels of these subunits were compared between cell lines by Two-way ANOVA with Dunnett’s correction for multiple comparisons. Each point on the graphs represents an individual organoid. Bars and error denote mean and SEM. * p < 0.05, **p < 0.01, ***p < 0.001, ****p < 0.0001.

**Additional file 16:** Age-dependent change in the production and release of neurotransmitters. (a, b) Heatmaps displaying the levels of neurotransmitters detected in the organoids (a) and media (b) at 3–4 months and 6–10 months old. Age-dependent changes in neurotransmitters were analysed by a repeated measures One-way ANOVA with Dunnett’s correction for multiple comparisons. (c) Immunofluorescence images of Parvalbumin-positive interneurons. The scale bar is 100 μm. * p < 0.05, **p < 0.01, ***p < 0.001, ****p < 0.0001.

**Additional file 17:** Expression levels of NKCC1 and NKCC2. Representative immunofluorescent images of the expression levels of NKCC1 and NKCC2 (red) in healthy control organoids (*HC*) and organoids with genetic mutations (*PRNP*^*E200K1*^, *PRNP*^*E200K2*^*, T21*, and *LRRK2*^G2019S^). The scale bar on the top left image indicates 500 μm.

**Additional file 18:** Levels of active caspase 3 before and after exposure to ZnCl_2_. (a) Representative immunofluorescence images of active caspase 3 in 6–10-month-old *HC* and dCOs before (basal) and after exposure to ZnCl_2_. Average fluorescence intensity of caspase 3 in a. The scale bar is 100 μm.

**Additional file 19:** Neuronal burst rate before and after the treatment with 0.5 µM pregnanolone.

## Data Availability

All data generated or analysed during this study are included in this published article [and its Additional files].

## Abbreviations

AD: Alzheimer’s disease
CJD: Creutzfeldt-Jakob-disease
CO: Cerebral organoid
dCOs: CO with disease-associated mutations
DS: Down syndrome
*HC*: Healthy controls
LFP: Local field potential
*LRRK2*: Leucine-Rich Repeat Kinase 2
*LRRK2*^*G2019S*^: *LRRK2* Mutation
MEA: Multielectrode arrays
“p” before neurotransmitter receptors: Phosphorylated receptors
PD: Parkinson’s disease
*PRNP*: Prion protein gene
*PRNP*^*E200K1*^*/PRNP*^*E200K2*^: E200K mutation within *PRNP* from donors 1 and 2.
RT‐QuIC: Real-time quaking-induced conversion
“s” before neurotransmitter receptors: Surface receptors
T21: Trisomy 21
