# Supplementary figures and images for: Neuronal excitatory-to-inhibitory balance is altered in cerebral organoid models of genetic neurological diseases
Source: Mol Brain. 2021 Oct 11;14:156. doi: 10.1186/s13041-021-00864-w (PMC8507222; doi:10.1186/s13041-021-00864-w)

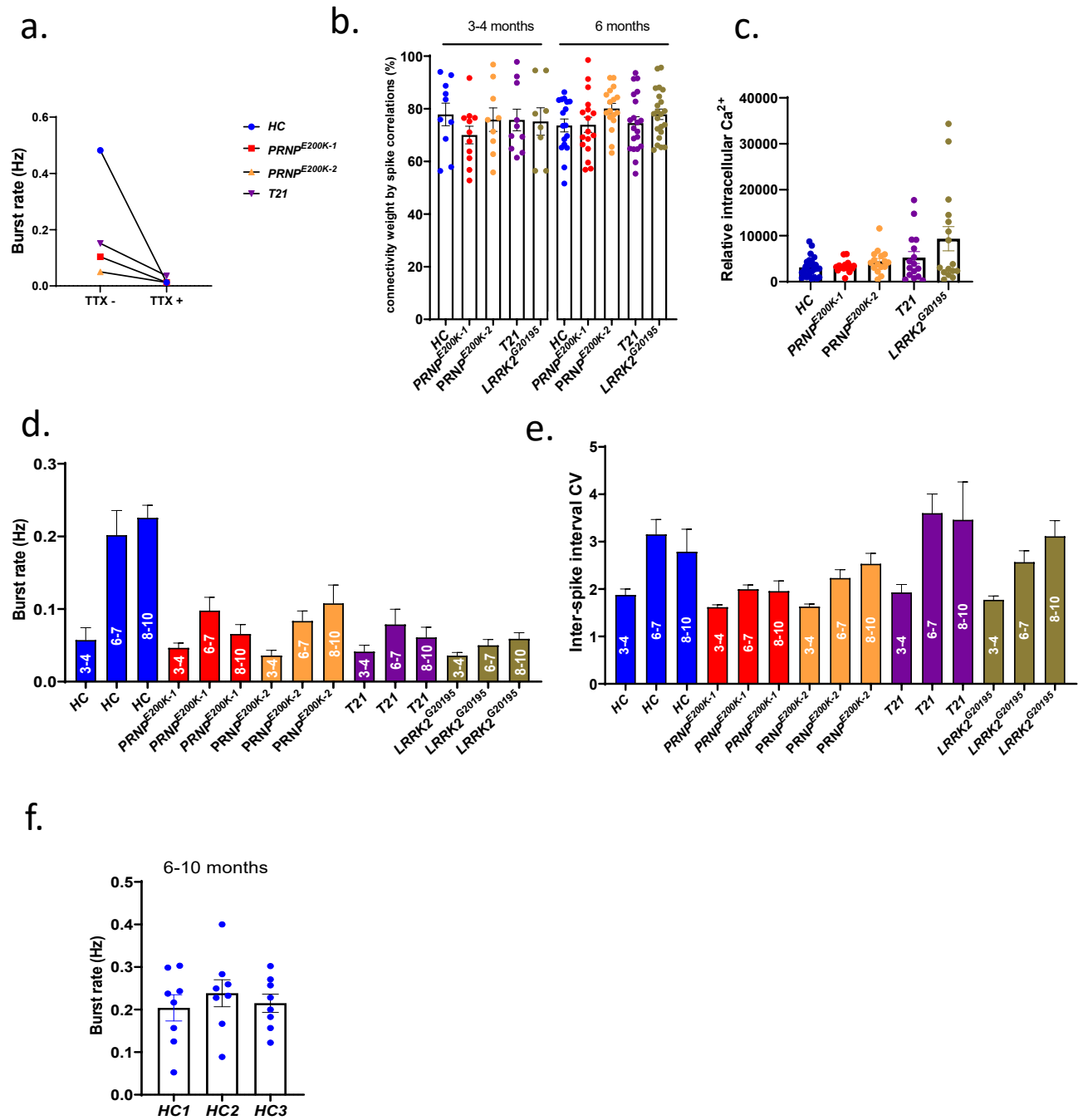

Supplement: Supplementary file 1 — Additional file 1: Neuronal firing and network communication. (a) Neuronal burst rate before and after exposure to TTX. (b) The strength (weight) of the connectivity between electrodes based on spike correlation in the healthy control organoids (HC) and organoids with genetic mutations (PRNPE200K1, PRNPE200K2, T21, and LRRK2G2019S) at 3–4 months (n = 8–11) and 6–10 (n = 17–21) months old. (c) Intracellular levels of calcium in all organoid lines at 6–10 months old (n = 16 for the HC and n = 28 in the other lines). (d, e) Burst rate (d) and inter-spike interval coefficient of variation (CV; e) in each organoid line at 3–4, 6–7, and 8–10 months old. (f) Burst rate of the three HC lines at 6–10 months old. Each point on the graphs represents an individual organoid. Bars and error denote mean and SEM. [file 13041_2021_864_MOESM1_ESM.pdf]

# Foliaki et al.\_Additional File 11

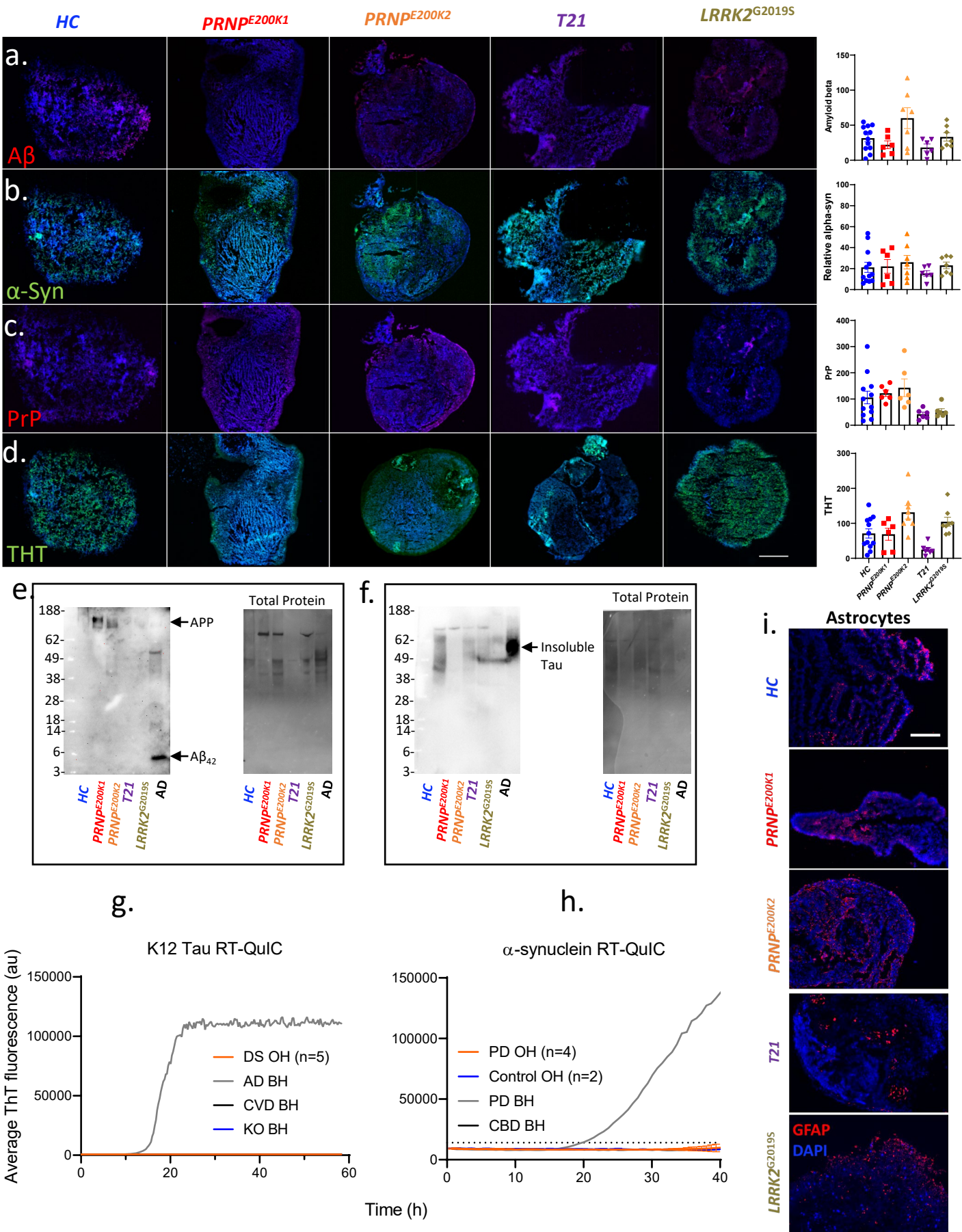

Supplement: Supplementary file 11 — Additional file 11: Assessing for disease-related pathology. Representative images showing immunofluorescence detection of total amyloid beta (a; antibody: 6E10), alpha-synuclein (b; antibody: anti-alpha-synuclein), PrP (c; antibody: EP1802Y), and THT (d) in 6 month old healthy controls (HC) and organoids with genetic defects (PRNPE200K1, PRNPE200K2,T21, LRRK2G2019S) as indicated. Scale bar is 500 µm. Corresponding plots (right) show pixel intensity quantification in DAPI-positive cells. (e) Western blot analysis of APP/amyloid beta in lysates from all the organoids and a brain homogenate from a terminal Alzheimer’s disease (AD) brain (antibody: 6E10). The arrows depict bands corresponding to the molecular mobility of APP and Aβ42. The right panel shows the total protein loaded for each sample. (f) A western blot analysis of insoluble Tau (solubilized by sarkosyl and pelleted by ultracentrifugation) in all the organoid lines and a brain homogenate from a terminal Alzheimer’s disease (AD) brain (antibody: T22). The right panel shows the total protein loaded for each sample. Bars and error denote mean and SEM. The levels of amyloid beta, alpha-synuclein, PrP, and THT were compared between organoid lines by One-way ANOVA on ranks with Dunnett’s correction for multiple comparisons. (g) Primary ThT fluorescence data of K12 Tau RT-QuIC. Representative traces from T21 tau-free mouse brain (KO), cerebrovascular disease (CVD), and Alzheimer disease (AD) brain homogenates, and Down syndrome (DS) organoid homogenates at 10–3 dilutions are displayed. Samples were also tested at 10–3-10–6 for CVD, 10–3-10–10 for AD, and 10–2-10–5 for DS. For the control brain homogenates, the curves are displayed as the average of 4 replicate wells. For the DS organoid homogenates, the curve is displayed as an average ± standard deviation of 5 independent organoids run in quadruplicate. (h) Primary ThT fluorescence data of α-synuclein RT-QuIC. Representative traces from Corticobasal degeneration [file 13041_2021_864_MOESM11_ESM.pdf]

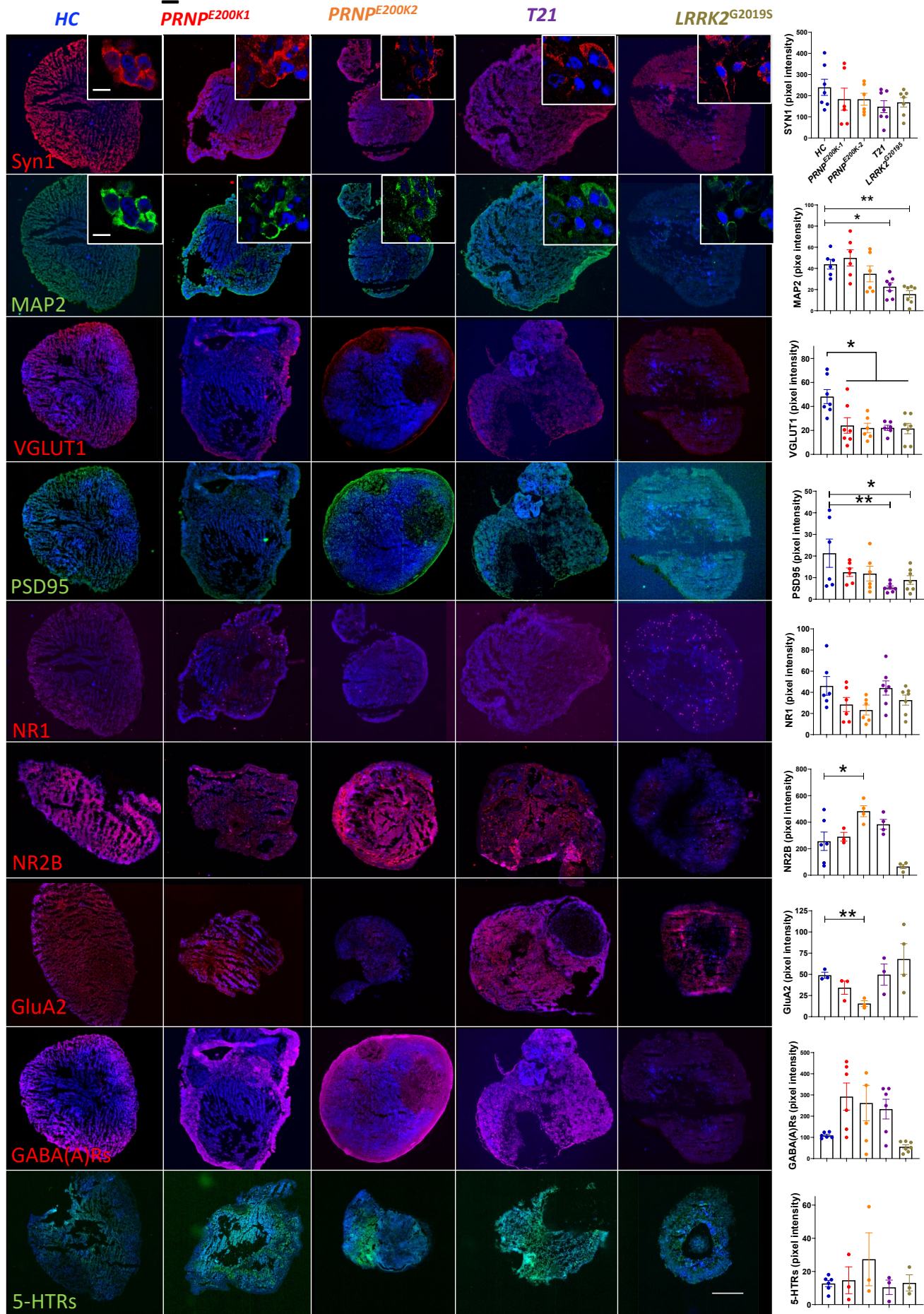

Supplement: Supplementary file 13 — Additional file 13: Total protein levels of key synaptic receptors. Representative immunofluorescence images showing detection of key synaptic proteins in 6–10-month-old healthy control organoids (HC) and organoids with genetic mutations (PRNPE200K1, PRNPE200K2, T21, and LRRK2G2019S) as indicated. Scale bar is 500 µm. The insets in the images of Syn1 and MAP2 show a high magnification images of these markers and their localization. Quantification of the pixel intensity of synaptic proteins (plots are shown right of the corresponding marker) in DAPI-positive cells was compared between organoid lines by One-way ANOVA on ranks with Dunnett’s correction for multiple comparisons. The quantification was done in the whole organoid section (imaged at 4 × magnification). Each point on the graphs represents an individual organoid. Bars and error denote mean and SEM* p < 0.05, **p < 0.01. [file 13041_2021_864_MOESM13_ESM.pdf]

Foliaki et al.\_Additional File 14

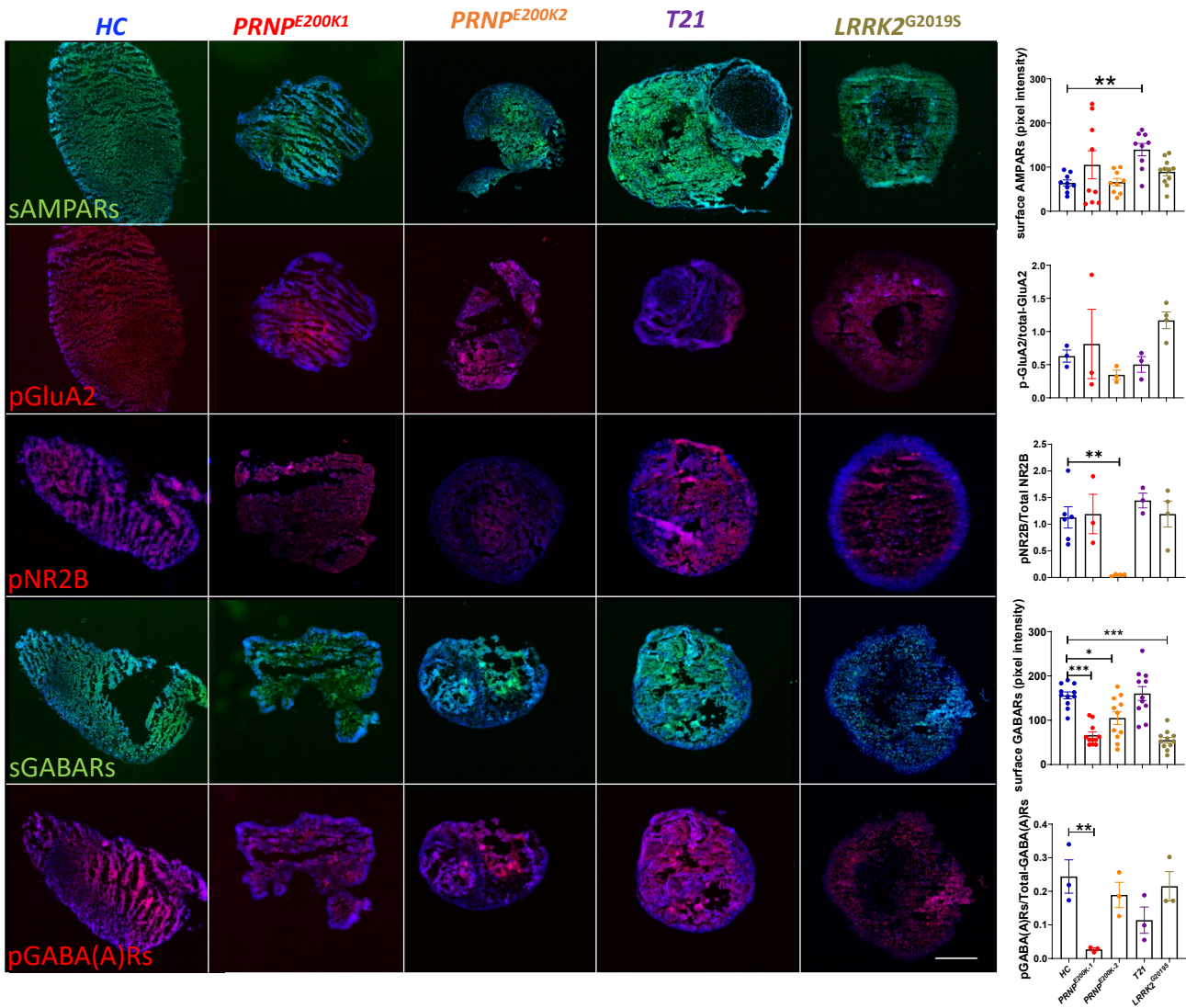

Supplement: Supplementary file 14 — Additional file 14: Expression levels of active synaptic proteins. Representative immunofluorescence images showing detection of surface AMPA receptors (sAMPARs), phosphorylated GluA2-containing AMPARs (pGluA2), phosphorylated NR2B-containing NMDA receptors (pNR2B), surface GABA receptors (sGABARs), and phosphorylated GABA(A) receptors (pGABA(A)Rs) in 6–10-month-old healthy control organoids (HC) and organoids with genetic mutations (PRNPE200K1, PRNPE200K2,T21, and LRRK2G2019S) as indicated. Quantification of pixel intensity (in DAPI-positive cells) of the surface AMPARs and GABARs and ratios of phosphorylated (active) GluA2, NR2B and GABA(A). Active protein detection was compared between organoid lines by One-way ANOVA on ranks with Dunnett’s correction for multiple comparisons. Each point on the graphs represents an individual organoid. Bars and error denote mean and SEM. Scale bar is 500 µm. * p < 0.05, **p < 0.01, ***p < 0.001. [file 13041_2021_864_MOESM14_ESM.pdf]

Foliaki et al.\_Additional File 15

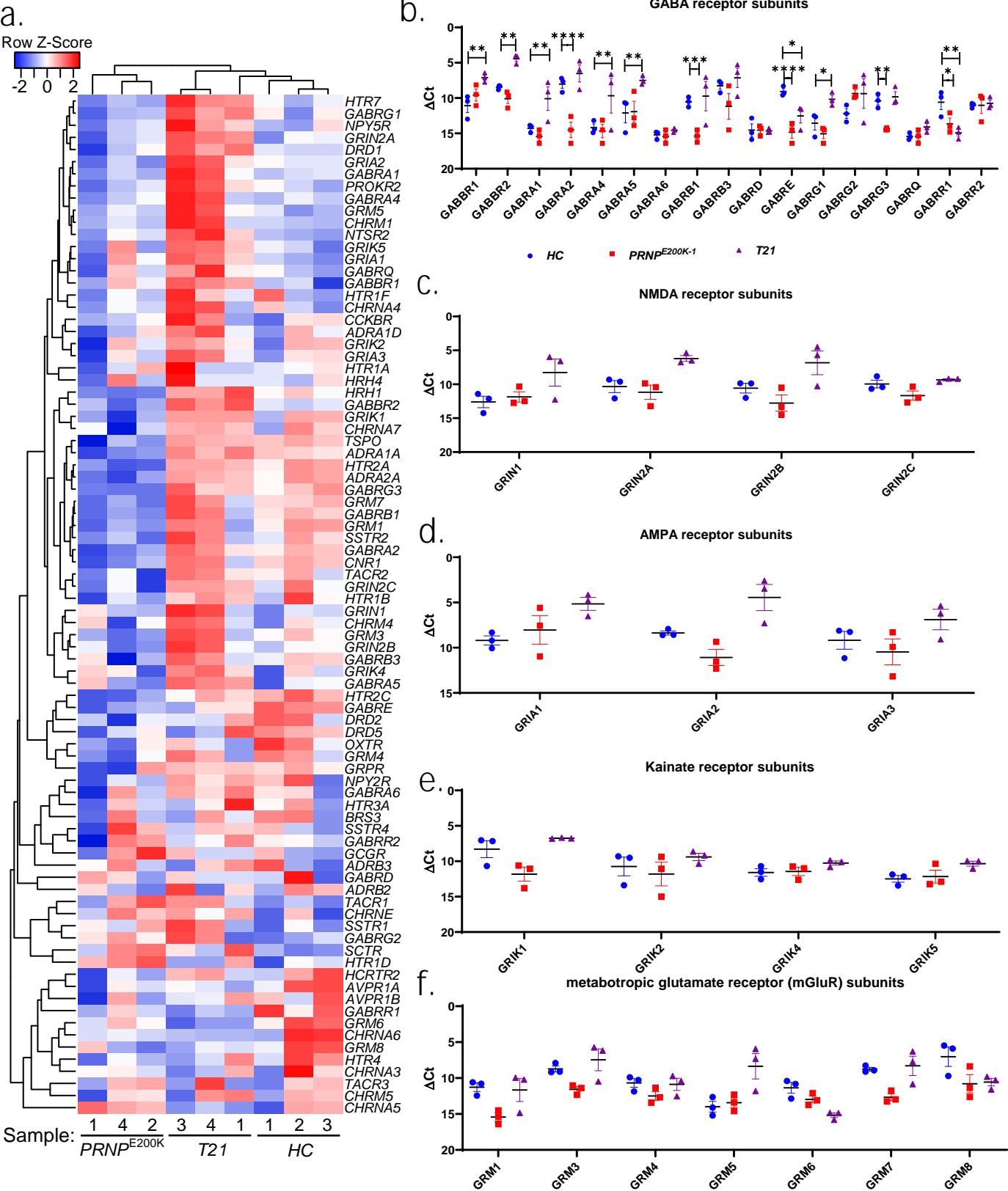

Supplement: Supplementary file 15 — Additional file 15: Expression of genes for neurotransmitter receptors at 6 months old. (a) Pearson correlation heatmap and average linkage hierarchical cluster analysis of the row Z-scores of the Delta Ct from the qRT-PCR analysis of various neurotransmitter receptors in 6–10-month-old healthy control organoids (HC) and organoids with genetic mutations (PRNPE200K1 and T21). (b-c) Some of the Delta Ct data presented in a, displaying the mRNA levels for the subunits of GABA receptors (b), NMDA receptors (c), AMPA receptors (d), kainate receptors (e), and metabotropic receptors (f; n = 3 per an organoid line). Expression levels of these subunits were compared between cell lines by Two-way ANOVA with Dunnett’s correction for multiple comparisons. Each point on the graphs represents an individual organoid. Bars and error denote mean and SEM. * p < 0.05, **p < 0.01, ***p < 0.001, ****p < 0.0001. [file 13041_2021_864_MOESM15_ESM.pdf]

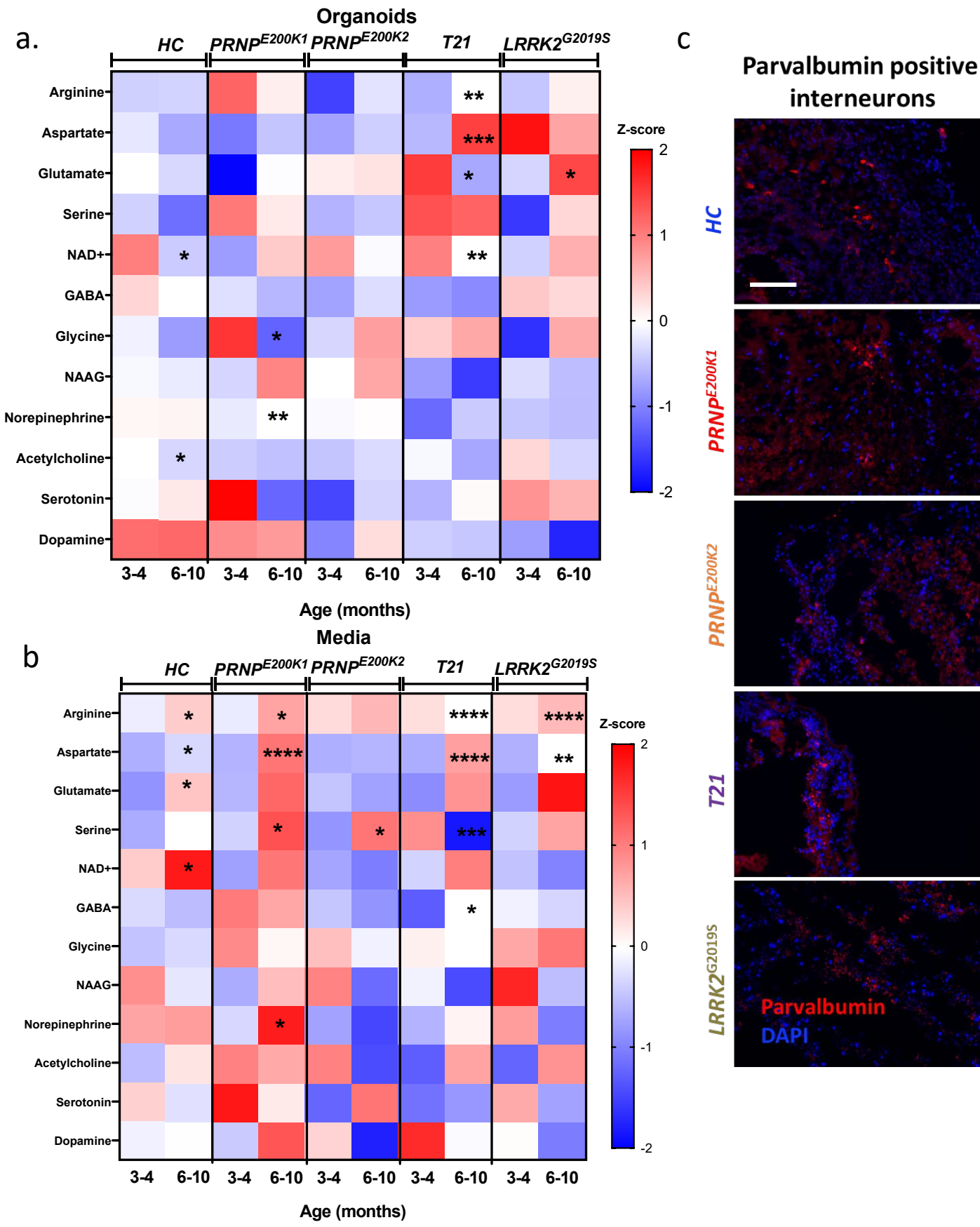

Supplement: Supplementary file 16 — Additional file 16: Age-dependent change in the production and release of neurotransmitters. (a, b) Heatmaps displaying the levels of neurotransmitters detected in the organoids (a) and media (b) at 3–4 months and 6–10 months old. Age-dependent changes in neurotransmitters were analysed by a repeated measures One-way ANOVA with Dunnett’s correction for multiple comparisons. (c) Immunofluorescence images of Parvalbumin-positive interneurons. The scale bar is 100 μm. * p < 0.05, **p < 0.01, ***p < 0.001, ****p < 0.0001. [file 13041_2021_864_MOESM16_ESM.pdf]

# Foliaki et al.\_Additional File 17

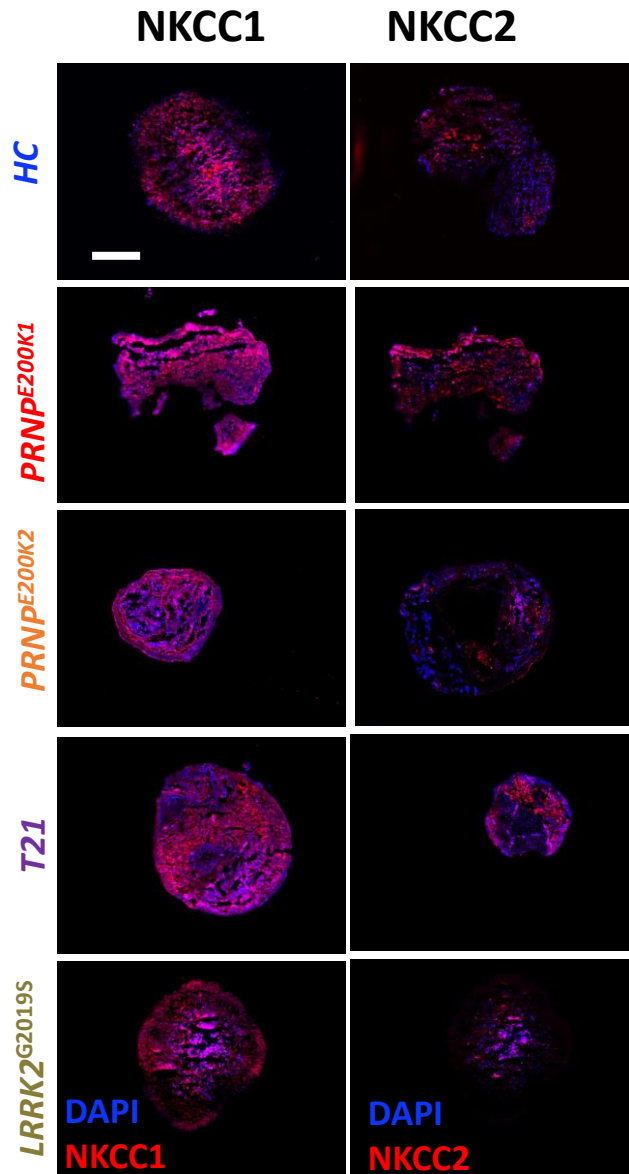

Supplement: Supplementary file 17 — Additional file 17: Expression levels of NKCC1 and NKCC2. Representative immunofluorescent images of the expression levels of NKCC1 and NKCC2 (red) in healthy control organoids (HC) and organoids with genetic mutations (PRNPE200K1, PRNPE200K2, T21, and LRRK2G2019S). The scale bar on the top left image indicates 500 μm. [file 13041_2021_864_MOESM17_ESM.pdf]

Foliaki et al.\_Additional File 18

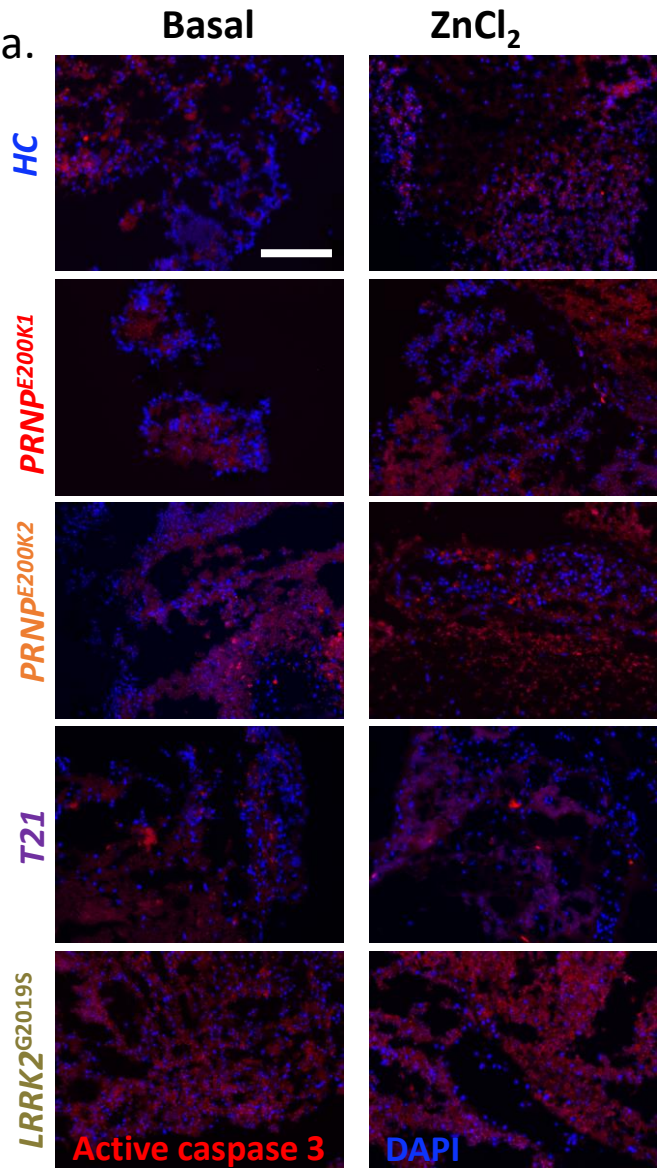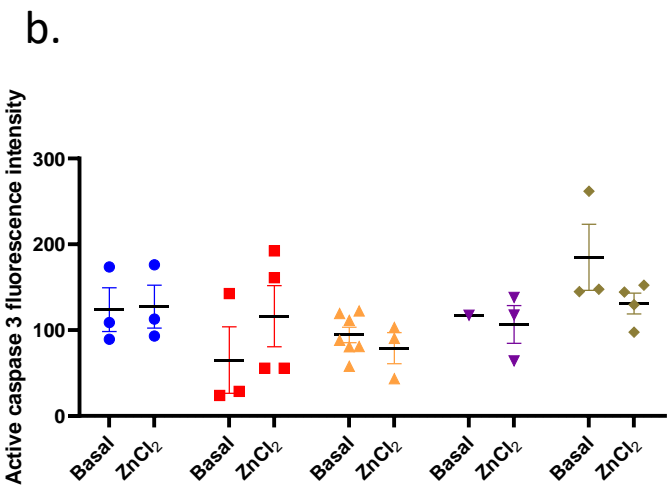

Supplement: Supplementary file 18 — Additional file 18: Levels of active caspase 3 before and after exposure to ZnCl2. (a) Representative immunofluorescence images of active caspase 3 in 6–10-month-old HC and dCOs before (basal) and after exposure to ZnCl2. Average fluorescence intensity of caspase 3 in a. The scale bar is 100 μm. [file 13041_2021_864_MOESM18_ESM.pdf]

# Foliaki et al. \_Additional File 19

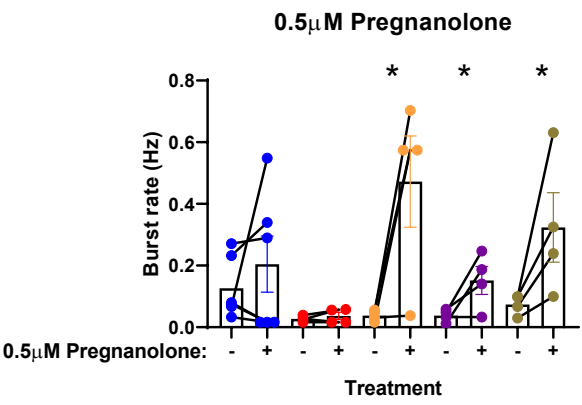

Supplement: Supplementary file 19 — Additional file 19: Neuronal burst rate before and after the treatment with 0.5 µM pregnanolone. [file 13041_2021_864_MOESM19_ESM.pdf]
