# Supplementary material for: Neuronal excitatory-to-inhibitory balance is altered in cerebral organoid models of genetic neurological diseases
Source: Mol Brain. 2021 Oct 11;14:156. doi: 10.1186/s13041-021-00864-w (PMC8507222; doi:10.1186/s13041-021-00864-w)
Supplement: Supplementary file 8 — Additional file 8: Primary antibody List. [file 13041_2021_864_MOESM8_ESM.pdf]

Foliaki et al._Additional File 8

Primary antibody list

Antibody	Company	Catalog number
Synapsin 1/2	Synaptic Systems	106 006
Anti-Vesicular Glutamate Transporter 1	Millipore Sigma	AB5905
MAP 2	Synaptic Systems	188 006
Anti-PSD95	Abcam	ab13552
GluN 1	Synaptic Systems	114 011
Anti-NMDAR2B	Abcam	ab65783
Anti-NMDAR2B (phospho S1303)	Abcam	ab8127
Anti-Ionotropic Glutamate receptor 2	Abcam	ab206293
Anti-Ionotropic Glutamate receptor 2 (phospho S880)	Abcam	ab52180
Anti-GABA A Receptor alpha 1	Abcam	ab33299
Anti-GABA A Receptor GABRB3 (phospho S408 + S409)	Abcam	ab254130
Anti-Serotonin antibody	Abcam	ab66047
Purified anti-β-Amyloid, 1-16 Antibody (6E10)	BioLegend	803004
Anti-Alpha-synuclein antibody	Abcam	ab6162
Anti-Prion protein PrP antibody [EP1802Y]	Abcam	ab52604
Anti-Tau (T22)	Millipore Sigma	ABN454
Anti-NKCC1 antibody	Abcam	ab59791
Anti-SLC12A1/NKCC2	Abcam	ab244342
Anti-Allopregnanolone antibody	Abcam	ab45164
Anti-Caspase-3 antibody	Abcam	ab13847
