# Supplementary material for: Neuronal excitatory-to-inhibitory balance is altered in cerebral organoid models of genetic neurological diseases
Source: Mol Brain. 2021 Oct 11;14:156. doi: 10.1186/s13041-021-00864-w (PMC8507222; doi:10.1186/s13041-021-00864-w)
Supplement: Supplementary file 9 — Additional file 9: Spectral information for neurotransmitters characterized on Sciex5500 QTRAP® mass spectrometer using ESI ionization. [file 13041_2021_864_MOESM9_ESM.pdf]

Foliaki et al._Additional File 9

Spectral information for neurotransmitters characterized on Sciex 5500 QTRAP® mass spectrometer using ESI ionization.

Compound	Polarity	Parent	Fragments				
**GABA**	negative	102	84	62	54	42	
**Norepinepherine**	positive	170	152.1	135	107.1		
	positive	152.1	135	107.1	79.1		
**Dopamine**	positive	154.1	137.1	109			
**Serotonin**	positive	177.1	160.1	142	132	115.1	105.2
	positive	160.1	142.1	132	115	105	
**NAAG**	negative	303.2	285.1	241.2	146.1	128.1	
**Acetylcholine**	positive	146.1	87.1	60.1	43.1		
