# Supplementary material for: Neuronal excitatory-to-inhibitory balance is altered in cerebral organoid models of genetic neurological diseases
Source: Mol Brain. 2021 Oct 11;14:156. doi: 10.1186/s13041-021-00864-w (PMC8507222; doi:10.1186/s13041-021-00864-w)
Supplement: Supplementary file 10 — Additional file 10: MRM information for molecular species targeted. DP: declusteringpotential, EP: entrance potential, CE: collision energy, CXP: collision cell exit potential. All source conditions are in volts. [file 13041_2021_864_MOESM10_ESM.pdf]

Foliaki et al._Additional File 10

MRM information for molecular species targeted. DP: declustering potential, EP: entrance potential, CE: collision energy, CXP: collision cell exit potential. All source conditions are in volts.

Negative						
Target	Q1	Q3	DP	EP	CE	CXP
Adenosine	266.4	134	-115	-10	-30	-17
ATP	506	408	-133.8	-14	-39	-42
ATP	506	159	-183	-14	-56	-49
Arginine	173	131	-125.3	-10	-19.1	-6.9
Arginine	173	156	-125.3	-10	-25	-6.9
Aspartate	132	88	-40.5	-10	-18.2	-9.2
Aspartate	132	115	-65.8	-10	-16.9	-5.9
Glutamate	146	128	-62.8	-10	-14.1	-8.6
Glutamate	146	102	-70.3	-10	-20	-7.4
Serine	104	74	-72.8	-10	-15.9	-8.7
Serine	104	42	-73.9	-10	-18.7	-12.2
NAD+	662	540	-42.1	-10	-26.7	-11.9
NAD+	662	79	-44.1	-10	-121.9	-9
NADH	664	408	-20	-10	-44.3	-16.3
NADH	664	79	-30	-10	-132.8	-12.7
GABA	102	84	-90	-10	-15	-40
GABA	102	54	-90	-10	-20	-25
Glycine	74	45	-120	-10	-20	-15
Glycine	74	58	-120	-10	-15	-15
NAAG	303.2	285.1	-50	-10	-15	-15
NAAG	303.2	128.1	-50	-10	-20	-15
Tryptophan	203	116	-57.9	-10	-20.9	-4.9
Tryptophan	203	74	-57.7	-10	-22	-12.7
Tyrosine	180	163	-66.4	-10	-18.1	-9.9
Tyrosine	180	119	-82.3	-10	-23.2	-10.7

Positive						
Target	Q1	Q3	DP	EP	CE	CXP
Adenosine	268.1	136	60	10	25	16
ATP	508	136.1	70	10	45	11
Arginine	175.1	70	50	10	32	9
Aspartate	134	69.9	40	10	23	8
Glutamate	148	83.9	40	10	22	10
Serine	106.1	59.9	50	10	15	9
NAD+	664.2	428.092	90	10	37	15
NADH	666.1	648.8	100	10	23	26
GABA	104	68.9	90	10	21	10
Norepinephrine	170.1	152.1	30	10	10	15
Norepinephrine	152.1	135.1	90	10	19	15
Norepinephrine	152.1	107.1	90	10	25	15
Acetylcholine	146.1	87.1	90	10	20	15
Acetylcholine	146.1	43.1	90	10	40	15
Serotonin	177.1	160.1	40	10	17	15
Serotonin	160.1	142.1	120	10	25	15
Dopamine	154.1	137.1	150	10	17	15
Dopamine	154.1	109.1	150	10	25	15
Tryptophan	205	146.1	50	10	25	9
Tyrosine	182	136.1	40	10	19	10
