# Supplementary material for: Neuronal excitatory-to-inhibitory balance is altered in cerebral organoid models of genetic neurological diseases
Source: Mol Brain. 2021 Oct 11;14:156. doi: 10.1186/s13041-021-00864-w (PMC8507222; doi:10.1186/s13041-021-00864-w)

# Foliaki et al._Additional File 12

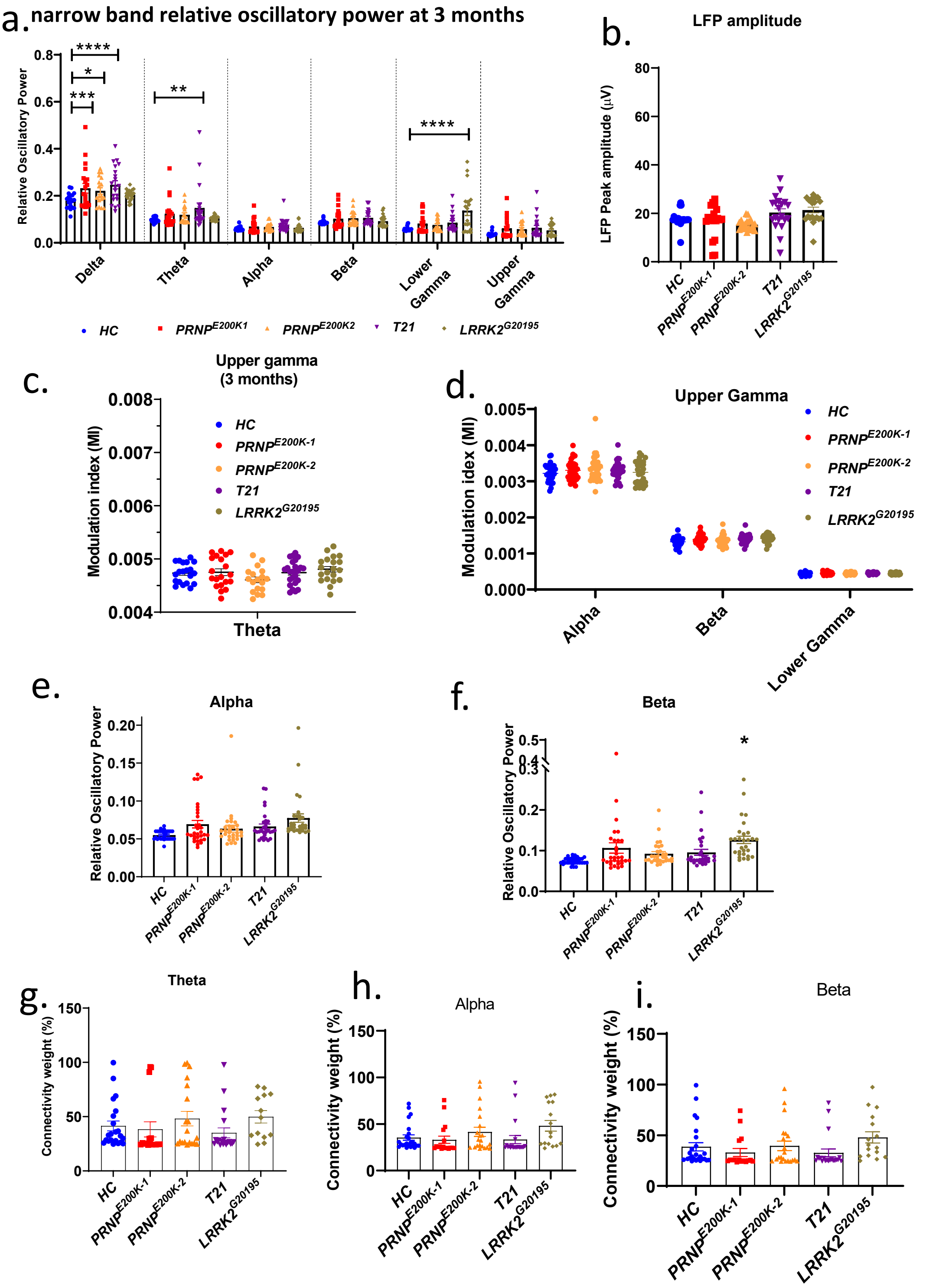

Supplement: Supplementary file 12 — Additional file 12: Neural oscillations at 3–4 months. (a) Narrow band oscillatory power in 3–4-month-old healthy controls (HC) and organoids with genetic mutations (PRNPE200K1, PRNPE200K2, T21, and LRRK2G2019S; n = 18 to 24). (b) Local field potential (LFP) peak amplitudes in all organoid lines at 3 months old. (c) The modulation index of the coupling between theta phase and the amplitudes of the upper gamma oscillations at 3 months old. (d) The modulation index of the coupling between the phases of alpha, beta, and lower gamma and the amplitudes of upper gamma oscillations in 3-month-old organoids. (e, f) Relative oscillatory power of alpha and beta bands in 3-month-old organoids. (g-i) The strength (weight/Pearson’s coefficient) of the connectivity between electrodes based on the correlation of oscillatory power. (a, d, e-i) Analysed by Two-way ANOVA with Dunnett’s correction for multiple comparisons. (b, c) Analysed by One-way ANOVA with Dunnett’s correction for multiple comparisons. Each point on the graphs represents an individual organoid. Bars and error denote mean and SEM. * p < 0.05, **p < 0.01, ***p < 0.001, ****p < 0.0001. [file 13041_2021_864_MOESM12_ESM.pdf]
